# Supplementary material for: Views of commissioners, managers and healthcare professionals on the NHS Health Check programme: a systematic review
Source: BMJ Open. 2017 Nov 15;7(11):e018606. doi: 10.1136/bmjopen-2017-018606 (PMC5695333; doi:10.1136/bmjopen-2017-018606)
Supplement: Author's manuscript [file bmjopen-2017-018606.draft_revisions.pdf]

BMJ Open

BMJ Open is committed to open peer review. As part of this commitment we make the peer review history of every article we publish publicly available.

When an article is published we post the peer reviewers' comments and the authors' responses online. We also post the versions of the paper that were used during peer review. These are the versions that the peer review comments apply to.

The versions of the paper that follow are the versions that were submitted during the peer review process. They are not the versions of record or the final published versions. They should not be cited or distributed as the published version of this manuscript.

BMJ Open is an open access journal and the full, final, typeset and author-corrected version of record of the manuscript is available on our site with no access controls, subscription charges or pay-per-view fees (http://bmjopen.bmj.com).

If you have any questions on BMJ Open's open peer review process please email editorial.bmjopen@bmj.com

BMJ Open

Views of commissioners, managers and healthcare professionals on the NHS Health Check programme: a systematic review

Journal:	*BMJ Open*
Manuscript ID	bmjopen-2017-018606
Article Type:	Research
Date Submitted by the Author:	09-Jul-2017

Complete List of Authors:	Mills, Katie; University of Cambridge, Public Health & Primary Care<br>Harte, Emma; RAND Europe<br>Martin, Adam; Academic Unit of Health Economics, Leeds Institute of Health Sciences<br>MacLure, Calum; RAND Europe<br>Griffin, Simon; The Primary Care Unit, Institute of Public Health<br>Mant, Jonathan; University of Cambridge, General Practice and Primary Care Research Unit<br>Meads, Catherine; Anglia Ruskin University, Faculty of Health, Social Care and Education<br>Saunders, Catherine; University of Cambridge, Cambridge Centre for Health Services Research<br>Walter, Fiona; University of Cambridge, Dept of Public Health and Primary Care<br>Usher-Smith, Juliet; The Primary Care Unit, Institute of Public Health

**Primary Subject Heading**:	Public health
Secondary Subject Heading:	Cardiovascular medicine, General practice / Family practice, Health policy, Qualitative research
Keywords:	NHS Health Check, health care professional views, systematic review

SCHOLARONE™
Manuscripts

Views of commissioners, managers and healthcare professionals on the NHS Health Check programme: a systematic review

Mills K[1], Harte E[2], Martin A[3], MacLure C[2], Griffin SJ[1,4], Mant J[1], Meads C[5], Saunders CL[1], Walter FM[1], Usher-Smith JA[1]

[1]The Primary Care Unit, Institute of Public Health, University of Cambridge, Box 113 Cambridge Biomedical Campus, Cambridge, CB2 0SR, UK

[2]RAND Europe, Westbrook Centre, Milton Road, Cambridge, CB4 1YG, UK

[3]Academic Unit of Health Economics, Leeds Institute of Health Sciences, University of Leeds, Leeds, LS2 9LJ, UK

[4]MRC Epidemiology Unit, University of Cambridge, Institute of Metabolic Science, Cambridge, CB2 0QQ, UK

[5]Faculty of Health, Social Care and Education, Anglia Ruskin University

Correspondence to: Juliet Usher-Smith jau20@medschl.cam.ac.uk

Key words: NHS Health Check, health care professional views, systematic review

Word count: 3987

For peer review only

ABSTRACT

Objective: To synthesize data concerning the views of commissioners, managers and health-care professionals towards the NHS Health Check programme in general and the challenges faced when implementing it in practice.

Design: A systematic review of surveys and interview studies with a descriptive analysis of quantitative data and thematic synthesis of qualitative data.

Data sources: An electronic literature search of Medline, Embase, Health Management Information Consortium (HMIC), Cumulative Index of Nursing and Allied Health Literature (CINAHL), Global Health, PsycInfo, Web of Science, OpenGrey, the Cochrane Library, NHS Evidence, Google Scholar, Google, Clinical Trials.gov and the ISRCTN registry to 09/11/16 with no language restriction and manual screening of reference lists of all included papers.

Inclusion criteria: Primary research reporting views of commissioners, managers or healthcare professionals on the NHS Health Check programme and its implementation in practice.

Results:

Of 18,524 citations, 15 articles met the inclusion criteria. There was evidence from both quantitative and qualitative studies that some commissioners and general practice healthcare professionals were enthusiastic about the programme while others raised concerns around inequality of uptake, the evidence-base and cost-effectiveness. In contrast, those working in pharmacies were all positive about programme benefits, citing opportunities for their business and staff. The main challenges to implementation were: difficulties with IT and computer software; resistance to the programme from some GPs; the impact on workload and staffing; funding; and training needs. Inadequate privacy was also a challenge in pharmacy and community settings, along with difficulty recruiting people eligible for Health Checks, and poor public access to some venues.

Conclusions:

The success of the NHS Health Check Programme relies on engagement by those responsible for its commissioning, management and delivery. Recognising and addressing the challenges identified in this review, in particular the concerns of GPs, is important for the future of the programme.

For peer review only

Strengths and limitations of this study

- This is the first study to systematically synthesize data concerning the views of commissioners, managers and healthcare professionals on the NHS Health Check programme.
- By including quantitative and qualitative data and studies not published in the mainstream medical literature it provides a comprehensive overview.
- However, the included studies were at risk of selection bias with recruitment consistently reported to have been difficult and all included only small sample sizes.
- Participants may also have responded in ways that reflected best practice or views they felt they ought to hold rather than their true views.

For peer review only

INTRODUCTION

Despite improvements in clinical care and reductions in risk factors such as smoking, cardiovascular disease (CVD) remains the leading cause of years of life lost in the UK[1], with nearly 400 people dying each day from CVD across England and Wales[2]. To help reduce this burden of disease the National Institute for Health and Care Excellence[3,4] and World Health Organization[5] recommend incorporating primary prevention initiatives. To address this, in 2009 Public Health England (PHE) introduced the NHS Health Check programme in England. The aim of the programme is to offer to all those between 40 and 74 years of age, with no pre-existing CVD, type 2 diabetes or dementia, an assessment of their risk of developing CVD and diabetes and advice about risk management, including medication, lifestyle advice and referral services.

The NHS Health Checks are held in General Practice (GP) surgeries, pharmacies, and community settings and are delivered by GPs, practice nurses, Health Care Assistants (HCAs), pharmacists and/or pharmacy assistants. Although it has been a mandated public health service since 2013 with clear guidelines on the required elements[6], there is flexibility in how local areas choose to commission the programme with GP surgeries and pharmacies choosing whether to deliver NHS Health Checks. The programme itself has also remained controversial, and its effectiveness has been challenged by both researchers and clinicians[7–9]. The result has been variability in approach to implementation and delivery across the country[10] and varying levels of engagement amongst health care professionals.

As with all individual-level interventions the impact of the NHS Health Check programme depends on those delivering it. This review synthesizes studies describing the views of commissioners, managers and healthcare professionals towards the NHS Health Check programme, and in doing so explores some of the reasons behind this variation and the challenges faced when implementing the programme.

METHODS

We performed a systematic literature review following a study protocol (available on request) that followed the PRISMA guidelines[11].

Search strategy

For peer review only

Published studies were identified from the results of an existing literature review conducted by PHE covering the period from 1st January 1996 to 9th November 2016. This was supplemented by a search in Web of Science and OpenGrey over the same time period. We undertook hand searches of the reference lists of all included publications and performed additional online searches for further publications by named authors identified in the search. Searches completed by PHE included the following sources: Medline, PubMed, Embase, Health Management Information Consortium (HMIC), Cumulative Index of Nursing and Allied Health Literature (CINAHL), Global Health, PsycInfo, the Cochrane Library, NHS Evidence, Google Scholar, Google, Clinical Trials.gov and the ISRCTN registry. Full details of all the search strategies are shown in Appendix 1. No language restrictions were applied.

Study eligibility criteria

Study selection was a two-part process. Initially, studies were screened by title and abstract for potential relevance to the NHS Health Checks. We excluded commentaries, editorials and opinion papers. In the second stage we identified studies reporting the views and experiences of healthcare professionals on NHS Health Checks. Two researchers (JUS and AM) read the full-text of all the potentially relevant studies. Studies for which it was unclear whether or not these inclusion criteria were met were discussed at consensus meetings with the wider research team.

Data extraction, quality assessment and synthesis

Data extraction was completed independently by two researchers (JUS + AM/CS/KM for the quantitative data and JUS + EH/CMa/KM for the qualitative data). Data extracted included study design, time period, recruitment method, participants and analytic method. Studies were also assessed for quality using the Critical Appraisal Skills Programme (CASP) checklist[12] for qualitative studies or a combined CASP checklist for cohort or randomized-controlled trials for the quantitative studies. No studies were excluded on the basis of quality alone.

We synthesized the qualitative data using thematic synthesis approaches which have been described in detail elsewhere[13]. Briefly, after initial reading and re-reading the papers we first coded all findings under the headings of “results” and findings” within the primary studies. We then organised these codes into descriptive, and subsequently analytical, themes. The initial coding was completed by two researchers (JUS and EH/CMa). Each researcher had

For peer review only

experience of conducting and analysing qualitative data and brought their own professional background (academic general practice, public services, health systems and innovation) to the interpretation of the findings. Consensus meetings were held with the wider research team, which included researchers with both clinical and non-clinical backgrounds and those with relevant topic expertise, to discuss the emerging codes and develop descriptive and analytical themes. To allow for appreciation of the data reviewed in these studies, illustrative quotations have been included alongside the analytical themes presented.

For the quantitative data, we extracted all the findings from the studies and synthesized those descriptively, grouping similar aspects together.

RESULTS

The initial literature search generated 18,524 titles and abstracts. 178 papers were potentially relevant to NHS Health Checks. These were reviewed at full text level (Figure 1). Of those, 164 were excluded. Reasons were that they did not include any relevant data for this research question, were duplicates or commentaries, or did not describe NHS Health Checks. Through citation searching one additional article was identified. This review is, therefore, based on 15 articles[14–28]. The characteristics of these are shown in Table 1 and the detailed quality assessment is shown in Tables 2a and b.

The studies used a range of designs. One included quantitative results from surveys[18], two quantitative results from surveys alongside free-text question responses[14,17,21], one free-text responses from a survey[28], and 10 findings from semi-structured interviews[15,16,19,20,22–27]. The majority (*n*=10) reported the views of healthcare professionals working within general practice. Four included pharmacists[16,18,20,22], four those delivering NHS Health Checks within community settings[22,24,25,28], and two commissioners[20,22]. Most collected data within the first two years of the programme (2009-2011). Sample sizes ranged between 25 and 442 for the survey studies and between 4 and 58 for the interview studies. All of the qualitative studies were considered to be of medium or high quality and the three quantitative studies were all of medium quality. Response rates for the two survey studies that reported them were 24% for GPs[17], 76% for practice managers[17], and 34% for pharmacists[18].

Overall views of the NHS Health Check Programme

For peer review only

Commissioners

Only one study reported the views of commissioners on the programmes as a whole[22]. Across the 14 commissioners interviewed, their enthusiasm for NHS Health Checks varied: while many approaching the programme positively, others described lower levels of engagement.

> "*It's very difficult to provide reassurance when on a personal level you're not sure if you've 100% bought into the programme either*" Commissioner[22]

General practice healthcare professionals

Two studies reported quantitative results from surveys with general practice healthcare professionals. In one, a survey of 43 GPs from 31 practices[17], 51% (*n*=22) viewed the programme as important, 54% (*n*=24) as beneficial to their patients, and 5% (*n*=2) considered the NHS Health Check programme to be a waste of time and resources. In the same study 36 out of 81 GPs and practice managers (44%) felt the high risk patient identification was beneficial to the practice. In a second survey of 25 healthcare professionals 72% (*n*=18) perceived that NHS Health Checks were useful in early detection and gave time to discuss patient health and lifestyles[14].

Of the ten interview studies, in general participants expressed the view that NHS Health Checks were beneficial in the early detection and prevention of disease[15,19,22,26].

> *"It's a good way to try and prevent illness and long term or serious conditions developing in the future"* Practice manager[19]

> *"I think it's a very good idea. We have a very high proportion of our patients who suffer with diabetes, almost 10% of our patients are diabetic so I thought this was an excellent opportunity to screen those earlier and pick them up."* GP[26]

There were, however, a number of concerns raised about the programme. In particular, some GPs described how they felt the programme attracted the "*worried well*" and that the patients who would benefit the most were the ones who were least likely to attend[14,15,21–23].

For peer review only

> *"if you send out an invite to a large number of people then the people who present themselves (laughs) er might well fit into that worried well category, um won't necessarily be um the HGV driver who works long hours and smokes a lot"* GP[23]

Many also described doubts about the long-term benefits and the costs of implementation, including staff resources and lack of evidence for the effectiveness[14,15,17,19,23].

> *"I don't think there is an awful lot of value. I think you'll pick up a few people a little bit earlier. Now whether that's worth the cost, obviously it's great for those individual patients, whether that's worth the cost of running a programme like this. I'd be amazed if it was."* Nurse[23]

> *"I think really this is mass screening and there's not a great deal of proof behind it.....Not entirely convinced with being told we have to offer a check to everyone."* GP[15]

Linked to this, participants in several of the studies described the challenges to achieving behaviour change and the difficulties they had getting people to make longstanding changes to their lifestyle following the health checks[26 15,25 14].

> *"Even if you access them, even if you find out that they're a really high risk score then getting these people to take on board you know the lifestyle changes, changes to their diet, exercising more. It's very difficult to get them to take those changes on."* Nurse[15]

Managing high-risk levels of alcohol consumption was felt to be especially challenging for some GPs and staff, particularly amongst patients in certain religious groups in which alcohol consumption can be stigmatised[26]. A lack of resources and lack of, or inconsistency of, well-funded support services in the wider community also contributed to this[14,15,22,26].

> *"We used to have things called exercise referral and we refer people to free gym sessions and send them to Slimming World and they'd get Slimming World sessions. We had really good responses and really good uptake for that, but that's all gone now."* Nurse[15]

For peer review only

Pharmacists

Three studies described the views of pharmacists[16,18,20]. Two of these interviewed pharmacists and those involved in the delivery of NHS Health Checks in pharmacies[16,18]. The third sent out a postal questionnaire to pharmacists, reporting a 34% response rate[20]. In contrast to the studies with healthcare professionals from general practice, very few participants from pharmacies discussed the benefits or otherwise of the NHS Health Checks to patients. Instead the focus was on the benefits of delivering NHS Health Checks in pharmacies, with all feeling it offered immense job satisfaction, promoted the image of the pharmacy and provided a good opportunity for staff development[16,18,20].

> "*I wanted to do this regardless... if I'm in a position where I can give somebody information that will then enable them to change their behaviour and live a healthier life that's a satisfying thing to do*." Pharmacist[16]

> *"For being the place to come in your local area for your health concerns, I think all round, for both the staff personally and for the company's goal, I think it's a positive thing."* Pharmacist[16]

Those delivering NHS Health checks in community settings

No studies reported the views towards the programme as a whole from those involved in delivering NHS Health Checks in community settings.

Challenges to implementation

One study reported challenges to implementation across all settings reported by commissioners[22]. In that study the greatest challenges were: engaging with GPs, both to encourage them to deliver NHS Health Checks within their practice and to facilitate delivery by non-GP providers; difficulties with data management in the absence of standard Read Codes when NHS Health Checks were first introduced, and the lack of clear national guidelines around data handling; and ensuring consistency of provision across GPs, particularly with the lack of a formal quality assurance or monitoring system at the time.

> *"The massive thing is the sheer variability in delivery. You get some star performers and some people that just won't engage with it"* Commissioner[22]

For peer review only - http://bmjopen.bmj.com/site/about/guidelines.xhtml

General practice

Seven studies described the challenges general practice healthcare professionals had experienced when implementing the NHS Health Checks within their practice. The main challenges are summarised in Table 3. Difficulties with IT and computer software were mentioned in over half of the studies, particularly related to the call and recall system when the programme was introduced[15,17,21,22] with 39% of practice managers in one study reporting difficulties with the clinical system, software, or errors in the existing data[17]. Impact on workload was also cited as a challenge for some. In a survey of 25 healthcare professionals, approximately 40% indicated there had been issues with staffing levels since starting to deliver NHS Health Checks, with some attributing these issues to the extra workload created by NHS Health Checks[14].

> *"NHS Health Check generates a huge workload for our staff in addition to what we do, a roughly 20 per cent additional workload*" Nurse[14]

In other studies, practice managers and GPs also generally agreed that the programme's impact on workload had knock-on effects on other services[17], with the financial reimbursement considered not sufficient to justify the work[17,22,26] or influencing their implementation[26].

> *"In order to get good payments we had to reach 50% target within three months …it was important for us to get the targets very very quickly*.' GP[26]

Concerns about remuneration were also reported by commissioners who claimed that NHS Health Checks were less of a priority as they are not part of the Quality and Outcomes Framework (QOF) for which GPs get paid[22].

> *"GPs have a very 'small business' mentality, and if the Health Care Assistant is off doing a Health Check and can't be doing something else for them, they get very jittery about that"* Commissioner[22]

Inadequate training was the final theme and was discussed in many of the studies[14,15,21,26]. These include a survey of 25 health care professionals in which 44% (*n*=11) indicated that

they required further training[14]. A survey of staff at 65 general practices in two inner London boroughs showed that staff at 62% (*n*=40) and 65% (*n*=42) of practices had attended training in delivering lifestyle advice or risk information, but only 43% (*n*=28) of practices reported that staff had attended training in measurement methods; at 23% (*n*=15) of practices no specific training was reported and 28% (*n*=18) considered that additional training would have been beneficial[21]. In free text responses 24% (*n*=5/21) of health care professionals suggested that improvements to staff training and capacity were required [21].

> *"[Training] would be good. As I say, we just learnt from our healthcare assistant what to do; basically it was like kind of on the job training… It would be nice to understand it in depth more, wouldn't it?"* HCA [15]

Pharmacies

Three studies reported the challenges faced by those involved in commissioning or delivering NHS Health Checks within pharmacies. In a survey of 442 community pharmacists[18], the three most important perceived barriers to implementation were lack of time, lack of staff and lack of reimbursement (all reported by over 55% of respondents). Lack of time and staff were also referred to in qualitative interviews with pharmacy staff. In particular, they described how, due to other commitments, most pharmacists did not have the capacity to perform the initial assessments as part of the NHS Health Checks. Instead, these were carried out by pharmacy assistants, who in turn needed more substantial training than was initially offered[16,18,20]

> "*The people they have working for them… haven't got the background in care knowledge or expertise. It wasn't like a GP surgery where you have Healthcare Assistants and Practice Nurses who on a day to day basis take blood pressures, take pulses, take blood and give advice on health*" PCT staff member[20]

Difficulties with funding were also discussed by commissioners who had had to develop different agreements from those with general practice as pharmacies pay VAT on all services they deliver and had had to allocate additional funds for unexpected costs, such as having to vaccinate pharmacy staff to allow them to handle blood and bodily fluids[20].

For peer review only

Other challenges (Table 3) identified by pharmacists and commissioners included: lack of private space for consultations (25% (n=111/442) [18]); difficulties with IT, particularly the need for a sufficiently secure internet connection to allow them to transfer patient identifiable data; and difficulty recruiting participants as the eligible population was largely dictated by footfall within the pharmacy[18,20,22]. Some pharmacies that were very close to GP practices delivering the NHS Health Check, also experienced competition between settings.

> *"Actually there's another problem, capturing the people. Everyone is out to capture them...it's very hard if you see someone coming in and say, 'Oh! You could be a candidate', and they say, 'The surgery has approached me and I'm going there'."* Pharmacy representative[20]

Community settings

Three studies reported the views of those involved in delivering NHS Health Checks in community settings[24,2822]. In contrast to some of the views expressed by HCAs working in general practice, in a small study of 10 HCAs delivering community-based NHS Health Checks, most felt there were enough staff and felt they had adequate support[28] and workers on a Health Bus found delivering NHS Health Checks to be fulfilling, enjoyable and overall a positive experience[22]. The main challenges identified (Table 3) were poor access to some venues, inadequate privacy, problems with some of the equipment and connection to the internet, and resistance from GPs to accept referrals from third-party providers.

> *"I don't think you come across very professional when you're sitting in a kitchen and all huddled round and all on top of each other. And it's not very nice for the patients, because…quite personal information"* Nurse[24]

> *"Because we were all in the same room it was easy to listen to what was happening next door."* HCA[28]

DISCUSSION

Key findings

While there was evidence that some commissioners, managers and healthcare professionals working in general practice could see the benefit of the NHS Health Check programme for patients, in the largest survey of GPs only half viewed the programme as important and

For peer review only

beneficial to their patients. A range of views was also seen in qualitative studies where some were enthusiastic while others raised concerns around inequality of uptake, the evidence behind the programme, and the cost-effectiveness. In contrast, those working in pharmacies were all positive about the programme, citing opportunities for their business and staff as reasons.

A number of challenges to implementation were identified. Difficulties with IT and computer software and resistance to the programme from GPs were described across all settings. The impact on workload and staffing, funding, and training needs were also challenges in general practice and pharmacy settings, while inadequate privacy was common to both pharmacies and community settings. Some pharmacies also experienced difficulty recruiting people for NHS Health Checks and poor access to some venues was reported in community settings.

Strengths and limitations

The strengths of this review include the comprehensive electronic search across multiple databases, the inclusion of reports not published within the mainstream medical literature, and the synthesis of both quantitative and qualitative data. However, all the studies included only small sample sizes and so may not be generalizable beyond the study context. In addition, recruiting GPs was consistently reported to have been difficult, especially from practices performing fewer NHS Health Checks, and the pharmacists who took part were all from pharmacies already involved in delivering NHS Health Checks; these studies are therefore at a particular risk of selection bias. Although the studies included a range of professionals from different settings, the views reported may, therefore, reflect the opinions of those who are particularly enthusiastic or negative, or have strong views about the NHS Health Check programme. The findings are also constrained by the questions addressed by the original researchers. Secondly, across all the studies it is possible that participants responded in ways that reflected best practice or the views they felt they ought to hold and so the findings may not reflect their true personal views. We also did not have access to the original data and so were only able to synthesize the findings considered by the authors of the original studies as worthy of report. Finally, all but two studies were conducted prior to 2013 and so may not reflect changes to the programme since then.

Comparison with existing literature

For peer review only

The main challenges to implementation identified in this study are consistent with those reported for prevention and health promotion in general. A multinational study across 11 European countries which included over 2000 GPs found that, although GPs believed prevention and health promotion was important, the workload, lack of time and need for funding limited their engagement[29]. Issues around workload and lack of time were also the two main barriers in a survey of GP views of their role in cancer prevention in the UK[30] and, along with lack of funding, were reported in a questionnaire survey of general practice healthcare professionals views on advising patients about physical activity[31] and a qualitative study of lifestyle counselling in Ireland[32]. The concerns expressed by some healthcare professionals in this study about the difficulties changing patients' behaviours are also commonly reported in the literature[31,33–35]: in one survey 40.3% (n = 112/278) GPs agreed that patients' behaviours are established and difficult to change[30].

Implications for clinicians, policymakers and future research

Given their central role in the success of the programme, the finding that a number of commissioners, GPs and other general practice staff had doubts about the evidence behind the programme has important implications for future delivery of NHS Health Checks. Lack of belief about proven effectiveness has been identified as one of the main barriers to offering health promotion activities within routine care amongst Dutch GPs and nurses[35] and evidence of effectiveness as one of the main incentives for GPs in a multinational study[29]. A survey of Australian GPs' views on clinical guidelines also cited an evidence base as the most important factor in their deciding whether to follow the recommendations of a guideline[36]. In the eight years since the programme was introduced there has been a growing evidence base around the NHS Health Checks. In contrast to the views held by many of the healthcare professionals in these studies, evidence suggests, for example, that, in part due to targeted approaches, more people in the most deprived quintile compared with the least deprived quintile have had NHS Health Checks[37–39] and there has been a consistent 3% to 4% increase in statin prescribing amongst attendees of the NHS Health Check compared with matched non-attendees[40–42]. Ensuring that programmes are effective and producing up-to-date, concise, summaries of the evidence and estimated benefits for different patient groups in an easily accessible format should therefore be a priority for those supporting delivery of the NHS Health Check programme and other similar prevention programmes. Piloting future programmes to provide such evidence before rolling them out nationally and including a phased roll out with in-built evaluation may also help address some of these concerns,

particularly amongst GPs whose engagement is key to delivery of the programme in all settings.

Anticipating and addressing training needs and difficulties with IT and computer software early may also increase engagement. Indeed, in 2013, since the majority of these studies were published, PHE introduced standard Read codes to facilitate data entry, updated software for identifying those eligible, and provided additional online training modules for healthcare professionals.

Overcoming some of the other challenges identified, such as funding and increased workload, are more difficult given the context of the current financial crisis within the NHS and reports of primary care services being stretched beyond safe limits by the needs of those with existing morbidity[43]. However, this review suggests there may be greater enthusiasm amongst pharmacies than general practices for delivering NHS Health Checks. Capitalising on this may be an effective way to reduce pressure on general practice whilst at the time empowering pharmacists to take on a wider role within healthcare[44].

ACKNOWLEDGEMENTS

We thank our patient and public representatives Kathryn Lawrence and Chris Robertson for providing helpful comments on the findings and the NHS Health Checks Expert Scientific and Clinical Advisory Panel working group for providing us with the initial literature search conducted by Public Health England. We would also like to thank Anna Knack, Research Assistant at RAND Europe, for her excellent research support, and Emma Pitchforth for her helpful comments on our analysis.

A summary of the findings reported in this manuscript have been published online by Public Health England (available at http://www.healthcheck.nhs.uk/commissioners_and_providers/evidence/) and RAND (http://www.rand.org/content/dam/rand/pubs/external_publications/EP60000/EP67129/RAND_EP67129.pdf). Permission from both has been obtained to publish the results in this journal.

Contributors

KM synthesised and interpreted the findings and wrote the first draft of the manuscript. EH screened articles for inclusion, extracted and synthesised the qualitative data, interpreted the findings and critically revised the manuscript. CMa extracted and synthesised the qualitative data and critically revised the manuscript. AM screened articles for inclusion, interpreted the findings and critically revised the manuscript. CS, CM, FW, SG and JM developed the protocol, interpreted the findings and critically revised the manuscript. JUS developed the protocol, screened articles for inclusion, extracted and synthesised the quantitative and qualitative data, interpreted the findings and wrote the first draft of the manuscript.

Funding

This work was funded by a grant from Public Health England. JUS and KM are funded by a Cancer Research UK/BUPA Foundation Cancer Prevention Fellowship (C55650/A21464) and FW by an NIHR Clinician Scientist award. The views expressed in this publication are those of the authors and not necessarily those of the NHS, the NIHR or the Department of Health.

All researchers were independent of the funding body and the funder had no role in data collection, analysis and interpretation of data; in the writing of the report; or decision to submit the article for publication.

Data sharing

All data are available from the reports or authors of the primary research. No additional data is available.

Competing Interests

All authors have completed the Unified Competing Interest form at *www.icmje.org/coi_disclosure.pdf* (available on request from the corresponding author) and declare that (1) they have no support from or relationships with companies that might have an interest in the submitted work in the previous 3 years; (2) their spouses, partners, or children have no financial relationships that may be relevant to the submitted work; and (3) they have no non-financial interests that may be relevant to the submitted work.

The corresponding author has the right to grant on behalf of all authors and does grant on behalf of all authors, an exclusive licence on a worldwide basis to the BMJ Publishing Group

For peer review only

Ltd and its Licensees to permit this article (if accepted) to be published in BMJ editions and any other BMJPGL products and sub-licences to exploit all subsidiary rights, as set out in our licence (http://resources.bmj.com/bmj/authors/checklists-forms/licence-for-publication).

All authors had full access to all of the data in the study and can take responsibility for the integrity of the data and the accuracy of the data analysis

The corresponding author affirms that the manuscript is an honest, accurate, and transparent account of the study being reported; that no important aspects of the study have been omitted; and that any discrepancies from the study as planned (and, if relevant, registered) have been explained.

FIGURE LEGENDS

Figure 1. PRISMA flow diagram

REFERENCES

1 Murray CJL, Richards MA, Newton JN, *et al.* UK health performance: findings of the Global Burden of Disease Study 2010. *Lancet (London, England)* 2013;**381**:997–1020.

2 Office for National Statistics Death Registrations. 2015.https://www.ons.gov.uk/peoplepopulationandcommunity/birthsdeathsandmarriages/deaths/datasets/deathregistrationssummarytablesenglandandwalesreferencetables (accessed 19 May2017).

3 National Institute for Health and Care Excellence. Cardiovascular disease prevention Public health guideline [PH25]. 2010.https://www.nice.org.uk/guidance/ph25 (accessed 19 May2017).

4 National Institute for Health and Care Excellence. Cardiovascular disease: risk assessment and reduction, including lipid modification Clinical guideline [CG181]. 2016.https://www.nice.org.uk/guidance/cg181 (accessed 19 May2017).

5 World Health Organisation. Package of Essential Noncommunicable interventions for primary health care in low-resource settings. www.who.int/nmh/publications/essential_ncd_interventions_lr_settings.pdf (accessed 19 May2017).

6 Public Health England. NHS Health Check Best practice guidance. Published Online First: 2015.www.healthcheck.nhs.uk/document.php?o=456

7 Capewell S, McCartney M, Holland W. Invited debate: NHS Health Checks-a naked emperor? *J Public Health (Oxf)* 2015;**37**:187–92.

8 Krogsbøll LT, Jørgensen KJ, Gøtzsche PC. General health checks in adults for reducing morbidity and mortality from disease. *JAMA* 2013;**309**:2489–90.

9 Krogsbøll LT, Jørgensen KJ, Grønhøj Larsen C, *et al.* General health checks in adults for reducing morbidity and mortality from disease: Cochrane systematic review and

meta-analysis. *BMJ* 2012;**345**:e7191.

10 Graley CEM, May KF, McCoy DC. Postcode lotteries in public health--the NHS Health Checks Programme in North West London. *BMC Public Health* 2011;**11**:738.

11 Moher D, Liberati A, Tetzlaff J, *et al.* Preferred reporting items for systematic reviews and meta-analyses: the PRISMA statement. *Int J Surg* 2010;**8**:336–41.

12 Critical Appraisal Skills Programme (CASP) Checklists. http://www.casp-uk.net/casp-tools-checklists

13 Thomas J, Harden A. Methods for the thematic synthesis of qualitative research in systematic reviews. *BMC Med Res Methodol* 2008;**8**:45.

14 Baker C, Loughren EA, Crone D, *et al.* Perceptions of health professionals involved in a NHS Health Check care pathway. *Pract Nurs* 2015;**26**:608–12.

15 Ismail H, Kelly S. Lessons learned from England's Health Checks Programme: using qualitative research to identify and share best practice. *BMC Fam Pract* 2015;**16**:144.

16 Crabtree V, Hall J, Gandecha M. NHS health checks: The views of community pharmacists and support staff. *Int J Pharm Pract* 2010;**18**:35–6.

17 Krska J, du Plessis R, Chellaswamy H. Views of practice managers and general practitioners on implementing NHS Health Checks. *Prim Health Care Res Dev* 2015;:1–8.

18 Loo RL, Diaper C, Salami OT, *et al.* The NHS Health Check: The views of community pharmacists. *Int J Pharm Pract* 2011;**19**:13.

19 McDermott L, Wright AJ, Cornelius V, *et al.* Enhanced invitation methods and uptake of health checks in primary care: randomised controlled trial and cohort study using electronic health records. *Health Technol Assess (Rockv)* 2016;**20**:1–92.

20 McNaughton RJ, Oswald NT, Shucksmith JS, *et al.* Making a success of providing NHS Health Checks in community pharmacies across the Tees Valley: a qualitative study. *BMC Health Serv Res* 2011;**11**:1–7.

21 Nicholas JM, Burgess C, Dodhia H, *et al.* Variations in the organization and delivery of the 'NHS health check' in primary care. *J Public Heal (United Kingdom)* 2013;**35**:85–91.

22 Research Works. Public Health England Understanding the implementation of NHS Health Checks. 2013.

23 Riley R, Coghill N, Montgomery A, *et al.* Experiences of patients and healthcare professionals of NHS cardiovascular health checks: A qualitative study. *J Public Health (Bangkok)* 2015;**38**:543–51.

24 Riley R, Coghill N, Montgomery A, *et al.* The provision of NHS health checks in a community setting: an ethnographic account. *BMC Health Serv Res* 2015;**15**:1–10.

25 Shaw RL, Pattison HM, Holland C, *et al.* Be SMART: examining the experience of implementing the NHS Health Check in UK primary care. *BMC Fam Pract* 2015;**16**:1.

26 Shaw RL, Lowe H, Holland C, *et al.* GPs' perspectives on managing the NHS Health Check in primary care: A qualitative evaluation of implementation in one area of England. *BMJ Open* 2016;**6**:e010951.

27 Oswald N, Mcnaughton R, Watson P, *et al.* Tees Vascular Assessment Programme Evaluation. 2010.

28 NHS Greenwich. Evaluation of NHS Health Check PLUS Community Outreach Programme in Greenwich. 2011;:1–61.

For peer review only

29 Brotons C, Björkelund C, Bulc M, *et al.* Prevention and health promotion in clinical practice: the views of general practitioners in Europe. *Prev Med (Baltim)* 2005;**40**:595–601.

30 McIlfatrick S, Keeney S, McKenna H, *et al.* Investigating the role of the general practitioner in cancer prevention: a mixed methods study. *BMC Fam Pr* 2013;**14**:58.

31 Douglas F, Torrance N, van Teijlingen E, *et al.* Primary care staff's views and experiences related to routinely advising patients about physical activity. A questionnaire survey. *BMC Public Health* 2006;**6**:138.

32 Lambe B, Collins C. A qualitative study of lifestyle counselling in general practice in Ireland. *Fam Pract* 2010;**27**:219–23.

33 Jallinoja P, Absetz P, Kuronen R, *et al.* The dilemma of patient responsibility for lifestyle change: perceptions among primary care physicians and nurses. *Scand J Prim Health Care* 2007;**25**:244–9.

34 Jacobsen ET, Rasmussen SR, Christensen M, *et al.* Perspectives on lifestyle intervention: the views of general practitioners who have taken part in a health promotion study. *Scand J Public Health* 2005;**33**:4–10.

35 Geense WW, van de Glind IM, Visscher TLS, *et al.* Barriers, facilitators and attitudes influencing health promotion activities in general practice: an explorative pilot study. *BMC Fam Pract* 2013;**14**:20.

36 Gupta L, Ward JE, Hayward RS. Clinical practice guidelines in general practice: a national survey of recall, attitudes and impact. *Med J Aust* 1997;**166**:69–72.

37 Forster AS, Dodhia H, Booth H, *et al.* Estimating the yield of NHS Health Checks in England: A population-based cohort study. *J Public Heal (United Kingdom)* 2015;**37**:234–40.

38 Chang K-M, Soljak M, Lee JT, *et al.* Coverage of a national cardiovascular risk assessment and management programme (NHS Health Check): Retrospective database study. *Prev Med (Baltim)* 2015;**78**:1–8.

39 Robson J, Dostal I, Sheikh A, *et al.* The NHS Health Check in England: an evaluation of the first 4 years. *BMJ Open* 2016;**6**:e008840.

40 Chang K, Lee J, Vamos E, *et al.* Impact of the National Health Service Health Check. *CMAJ* 2016;**188**:E228-238.

41 Forster AS, Burgess C, Dodhia H, *et al.* Do health checks improve risk factor detection in primary care? Matched cohort study using electronic health records. *J Public Health (Bangkok)* 2015;:1–8.

42 Robson J, Dostal I, Madurasinghe V, *et al.* NHS Health Check comorbidity and management : an observational matched study in primary care. *Br J Gen Pract* Published Online First: 2016. doi:10.3399/bjgp16X688837

43 The Kings Fund. Understanding pressures in general practice. 2016.http://www.kingsfund.org.uk/sites/files/kf/field/field_publication_file/Understanding-GP-pressures-Kings-Fund-May-2016.pdf

44 Royal Pharmaceutical society. Now or never: Shaping pharmacy for the future. 2013.https://www.rpharms.com/resources/reports/now-or-never-shaping-pharmacy-for-the-future

For peer review only

Table 1. Features of studies

Author/ year	Type of report	Study period	Location of study	Setting of NHS Health Check	Data collection method	*n*	Method of recruitment to study	Participant characteristics	Method of analysis
Baker 2015[14]	Journal article	Not given	South West England	30.1% of total practices delivering NHS Health Checks	Surveys including quantitative and qualitative questions	25	Identified randomly via the County Medical List to ensure geographic spread	2 GPs, 14 practice managers, 6 practice nurses, 2 healthcare assistants and 1 administrator	Descriptive statistics Thematic analysis
Crabtree 2010[16]	Conference abstract	2009	Not given	32 (of 35) pharmacies in the area delivering NHS Health Checks	Semi-structured telephone interviews	32	All 35 pharmacies delivering the service were contacted	15 pharmacists, 13 support staff and 4 pre-registration pharmacists	Thematic analysis
Greenwich 2011[28]	Report	2011	Greenwich	Community	Open-ended questionnaire	11	All (12) clinicians delivering community outreach services providing NHS Health Checks were invited	Healthcare assistants, nurses, pharmacists and health trainers	Not described
Ismail and Kelly 2015[15]	Journal article	2010	Yorkshire	25 general practices	Semi-structured interviews	58	Letters of invitation or flyers to 41 general practices targeted to reflect diversity in terms of performance	Healthcare assistants, GPs, practice managers, practice nurses and other support staff	Framework analysis
Krska 2015[17]	Journal article	2011	Sefton, an area of North West England	33 (of 55) general practices	Postal survey with free text responses	83 (76% of practice managers, 24% of GPs)	Personally addressed letters of invitation with a covering letter to all practice managers and GPs at 55 practices	40 practice managers and 43 GPs	Categorisation of responses
Loo 2011[18]	Conference abstract	2009	Not given	Pharmacies	Postal questionnaire	442 (34%)	Questionnaire posted to all pharmacies in the area	All pharmacists 59% male; 89.1% full time; 53.4% worked for large multiple pharmacies	Descriptive statistics
McDermott 2016[19]	Journal article	2013-15	2 London boroughs	17 general practices	Semi-structured interviews	24	Recruited from within a trial of an enhanced invitation method	52% practice managers, 9% healthcare assistants, 30% administrators, 9% public health leads	Framework analysis
McNaughton 2011[20]	Journal article	Not given	Tees Valley	8 pharmacies	Semi-structured interviews	20	Postal invitation	10 primary care trust members, 8 pharmacists, 2 representatives from Local Pharmaceutical Committee	Thematic analysis
Nicholas 2012[21]	Journal article	2011	2 London boroughs	70 (of 96) general practices	Survey with free text responses	65	Invitations to all 96 general practices	25 practice managers, 8 GPs, 16 practice nurses, 2 healthcare assistants, 3	Descriptive statistics Content

								administrators and 14 not specified	analysis
Oswald 2010[27]	Evaluation report	2009 – 2010	Teesside	13 general practices	Semi-structured interviews	25	Letter of invitation to practice managers	8 practice managers, 14 practice nurses, 1 GP, 1 healthcare assistant, 1 pharmacist	Thematic analysis
Research works 2013[22]	Research report	2013	Not given	All settings	Semi-structured interviews	26	Contacts provided by Commissioners with snowballing recruitment	14 commissioners, 12 GPs, practice managers, health care assistant, nurse practitioner, physical activity development officer, health bus workers and a community pharmacist	Not described
Riley 2015[24]	Journal article	2013	Bristol inner-city	Community settings	Semi-structured interviews	4	Participants were recruited via their involvement with community outreach events.	1 practice nurse, 1 healthcare assistant, 1 engagement worker and 1 health trainer	Thematic analysis
Riley 2015[23]	Journal article	2013-14	Bristol	11 general practices	Semi-structured interviews	15	18 were invited with purposive sampling	5 GPs, 5 practice nurses, 3 healthcare assistants, 2 pharmacists	Thematic analysis
Shaw 2015[25]	Journal article	2010-11	Birmingham and Black Country	General practices and community	Semi-structured interviews	31	Recruited through lead clinicians	9 GPs, 6 practice managers, 4 practice nurses, 6 healthcare assistants, 1 alternative provider director, 1 call centre manager, 2 call centre operatives and 2 alternative provider registered practice nurses	Thematic analysis
Shaw 2016[26]	Journal article	Not given	Birmingham	General practices	Semi-structured interviews	9	Recruitment undertaken by local NHS trust. No further details provided	All GPs	Thematic analysis

Table 2a. Quality assessment of studies including surveys

Author, date	Study addressed a clearly focused issue	Use of an appropriate method / Randomisation (for RCTs)	Recruitment / comparability of study groups at baseline	Blinding (for RCTs)	Exposure measurement	Outcome measurement	Comparability of study groups during study (for RCTs)	Follow up (for longitudinal studies)	Confounding factors (for non-RCTs):	Applicability to England	Overall
Baker 2015[14]	⬤	●	●	n/a	n/a	●	n/a	n/a	•	•	Medium
Krska 2015[17]	⬤	⬤	●	n/a	n/a	●	n/a	n/a	•	●	Medium
Loo 2011[18]	⬤	⬤	●	n/a	n/a	n/a	n/a	n/a	•	●	Medium

• Low ● Medium ⬤ High

peer review only

Table 2b. Quality assessment of studies including qualitative data

Author, date	Study addressed a clearly focused issue	Appropriateness of qualitative method	Design	Recruitment	Consideration of relationship between research and participants	Ethical issues	Rigor of data analysis	Clarity of statement of findings	Overall
Baker 2015[14]	High	High	High	Low	Low	Medium	Medium	High	Medium
Crabtree 2010[16]	High	High	Low	Low	Low	Medium	Low	Medium	Medium
Greenwich 2011[28]	High	High	High	Medium	Low	Low	Low	Medium	Medium
Ismail and Kelly 2015[15]	High	High	High	Medium	Medium	High	High	High	High
Krska 2015[17]	High	High	Medium	High	n/a	Medium	Medium	Medium	Medium
McDermott 2016[19]	High	High	High	Low	Low	Medium	Medium	Medium	Medium
McNaughton 2011[20]	High	High	High	High	Low	High	High	High	High
Nicholas 2013[21]	High	High	High	High	n/a	Low	High	High	High
Oswald 2010[27]	High	High	High	Medium	Low	Medium	Medium	Medium	Medium
Research Works 2013[22]	High	High	High	Medium	Low	Low	Low	Medium	Medium
Riley 2015[23]	High	High	High	Medium	Medium	High	High	High	High
Riley 2015[24]	High	High	High	Medium	Medium	High	High	High	High
Shaw 2015[25]	High	High	High	Medium	Low	High	High	High	High
Shaw 2016[26]	High	High	High	Medium	Low	Medium	High	High	High

• Low ● Medium ⬤ High

Table 3. Challenges to implementation of NHS Health Checks reported across the settings

**Challenge to implementation**	**General practices**	**Pharmacies**	**Community settings**
Difficulties with IT and computer software	✓[15,17,21,22]	✓[20,22]	✓[22,24,28]
Impact on workload / staffing	✓[14,15,17,21]	✓[16,18,20]	
Funding	✓[15 17]	✓[18,20]	
Training needs	✓[14,15,21,26]	✓[18,20]	
Resistance from GPs	✓[22]	✓[22]	✓[22]
Inadequate privacy		✓[18,20,22]	✓[24,28]
Difficulty recruiting participants		✓[20,22]	
Poor access to some venues			✓[28]

For peer review only

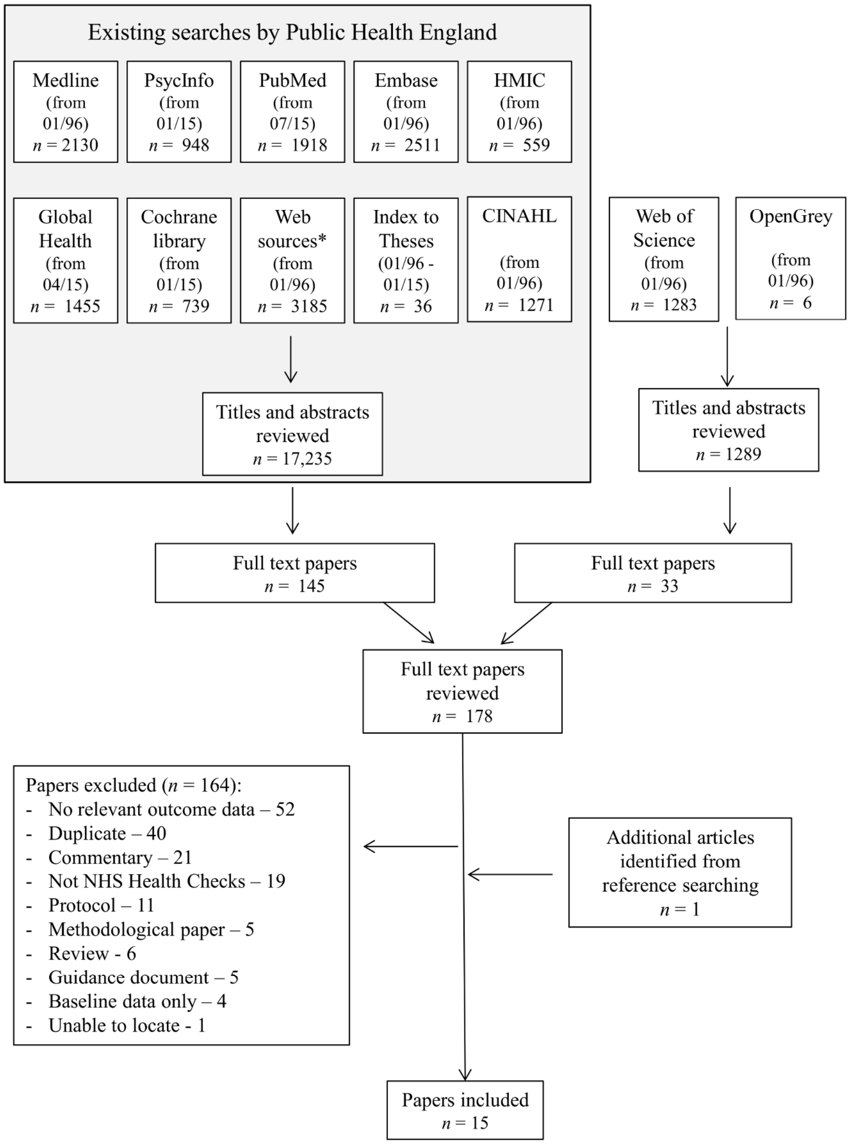

PRISMA flow diagram

190x254mm (300 x 300 DPI)

Appendix 1 – Search strategies

Database	Search strategy
Ovid Medline	1. health check*.tw.<br>2. (diabetes adj3 screen*).tw.<br>3. (cardiovascular adj3 screen*).tw.<br>4. (population adj2 screen*).tw.<br>5. (risk factor adj3 screen*).tw.<br>6. (opportunistic adj3 screen*).tw.<br>7. medical check*.tw.<br>8. general check*.tw.<br>9. periodic health exam*.tw.<br>10. annual exam*.tw.<br>11. annual review*.tw.<br>12. NHSHC.tw.<br>13. 1 or 2 or 3 or 4 or 5 or 6 or 7 or 8 or 9 or 10 or 11 or 12<br>14. cardiovascular adj3 prevention.tw.<br>15. (primary care or general practice or primary healthcare).tw<br>16. 14 and 15<br>17. Cardiovascular Diseases/ AND Primary Prevention/<br>18. 16 or 17<br>19. 13 or 18
PubMed	1. health check*<br>2. diabetes screen*<br>3. cardiovascular screen*<br>4. population screen*<br>5. risk factor screen*<br>6. opportunistic screen*<br>7. medical check*<br>8. general check*<br>9. periodic health exam*<br>10. annual exam*<br>11. annual review*<br>12. NHSHC<br>13. 1 or 2 or 3 or 4 or 5 or 6 or 7 or 8 or 9 or 10 or 11 or 12<br>14. Cardiovascular Diseases AND Primary Prevention[MeSH Terms]<br>15. "primary care"[Text Word] OR "general practice"[Text Word] OR "primary healthcare"[Text Word])<br>16. (cardiovascular[Text Word] AND prevention[Text Word])<br>17. #15 and #16<br>18. #14 or #17<br>19. #13 or #18
Ovid Embase	1. health check*.tw.<br>2. (diabetes adj3 screen*).tw.<br>3. (cardiovascular adj3 screen*).tw.<br>4. (population adj2 screen*).tw.

For peer review only

	5. (risk factor adj3 screen*).tw.<br>6. (opportunistic adj3 screen*).tw.<br>7. medical check*.tw.<br>8. general check*.tw.<br>9. periodic health exam*.tw.<br>10. annual exam*.tw.<br>11. annual review*.tw.<br>12. NHSHC.tw.<br>13. periodic medical examination/<br>14. 1 or 2 or 3 or 4 or 5 or 6 or 7 or 8 or 9 or 10 or 11 or 12 or 13<br>15. cardiovascular adj3 prevention.tw.<br>16. (primary care or general practice or primary healthcare).tw<br>17. 15 and 16<br>18. cardiovascular disease/ AND primary prevention/<br>19. 17 or 18<br>20. 14 or 19
Ovid HMIC	1 "health check*".af.<br>2 health checks/<br>3 (cardiovascular or vascular or heart or diabetes or stroke).af.<br>4 (screen* or risk).af.<br>5 3 AND 4<br>6 1 OR 2 or 5<br>7 cardiovascular adj3 prevention.tw.<br>8 (primary care or general practice or primary healthcare).tw<br>9 7 and 8<br>10 Cardiovascular diseases/ AND exp preventive medicine/<br>11 9 or 10<br>12 6 or 11
EBSCO CINAHL	S10 S1 OR S2 OR S9<br>S9 S5 OR S8<br>S8 S6 AND S7<br>S7 (MH "Preventive Health Care+")<br>S6 (MH "Cardiovascular Diseases+")<br>S5 S3 AND S4<br>S4 "primary care" or "general practice" or "primary healthcare"<br>S3 TX cardiovascular N3 prevention<br>S2 (diabetes N3 screen*) OR (cardiovascular N3 screen*) OR (population N2 screen*) OR (risk factor N3 screen*) OR (opportunistic N3 screen*) OR “medical check*” OR “general check*” OR “periodic health exam*” OR “annual exam*” OR "annual review*" OR NHSHC<br>S1 health check*
EBSCO Global Health	S10 S6 OR S19 OR S3 Limiters - Publication Year: 2016<br>S9 S7 AND S8<br>S8 DE "preventive medicine"<br>S7 DE "cardiovascular diseases"<br>S6 S4 AND S5<br>S5 "primary care" or "general practice" or "primary healthcare"

For peer review only

	S4 TX cardiovascular N3 prevention<br>S3 S1 OR S2<br>S2 (diabetes N3 screen*) OR (cardiovascular N3 screen*) OR (population N2 screen*) OR (risk factor N3 screen*) OR (opportunistic N3 screen*) OR “medical check*” OR “general check*” OR “periodic health exam*” OR “annual exam*” OR "annual review*" OR NHSHC<br>S1 health check*
HDAS PsycInfo	1 "health check*".af.<br>2 PHYSICAL EXAMINATION/<br>3 HEALTH SCREENING/<br>4 "diabetes screen*".af<br>5 "cardiovascular screen*".af<br>6 "population screen*".af<br>7 ("opportunistic* screen*" OR "risk factor screen*").af<br>8 ("medical check*" OR "general check*" OR "periodic health exam*" OR "annual exam*" OR "annual review*" OR NHSHC).af<br>9 1 OR 2 OR 3 OR 4 OR 5 OR 6 OR 7 OR 8<br>10 cardiovascular.ti,ab<br>11 prevention.ti,ab<br>12 10 AND 11<br>13 CARDIOVASCULAR DISORDERS/<br>14 PREVENTIVE MEDICINE/<br>15 13 AND 14<br>16 12 OR 15<br>17 9 OR 16
Web of Science, Science Citation Index	“health check*” OR “diabetes screen*” OR “cardiovascular screen*” OR “population screen*” OR “risk factor screen*” OR “Opportunistic screen*” OR “medical check*” OR “general check*” OR “periodic health exam*” OR “annual exam*” OR “annual review*” OR NHSHC<br>OR<br>(Cardiovascular NEAR/3 prevention) AND (“primary care” OR “general practice” OR “primary healthcare”)<br>Limit to: England, Scotland, Wales, North Ireland
Cochrane Library (Wiley)	#1 "health check*"<br>#2 (diabetes next/3 screen*) or (cardiovascular next/3 screen*) or (population next/2 screen*) or (opportunistic next/2 screen*) or ("risk factor" next/3 screen*) or "medical check*" or "general check*" or "periodic health exam*" or "annual exam*" or "annual review*" or NHSHC<br>#3 cardiovascular adj3 prevention.tw.<br>#4 (primary care or general practice or primary healthcare).tw<br>#5 #3 and #4<br>#6 MeSH descriptor: [Cardiovascular Diseases] this term only<br>#7 MeSH descriptor: [Primary Prevention] explode all trees<br>#8 #6 and #7<br>#9 #5 or #8<br>#10 #1 or #2 or #9

For peer review

NHS Evidence	“health check*” OR cardiovascular prevention primary care
TRIP database	“health check*” OR cardiovascular prevention primary care
Google Scholar	"nhs health check"<br>cardiovascular “health check”<br>cardiovascular prevention “primary care”
Google	"nhs health check"<br>cardiovascular prevention “primary care”<br>cardiovascular “health check”
Clinical trials.gov and ISRCDN registry	“health check”

For peer review only

PRISMA 2009 Checklist

Section/topic	#	Checklist item	Reported on page #
**TITLE**			
Title	1	Identify the report as a systematic review, meta-analysis, or both.	1
**ABSTRACT**			
Structured summary	2	Provide a structured summary including, as applicable: background; objectives; data sources; study eligibility criteria, participants, and interventions; study appraisal and synthesis methods; results; limitations; conclusions and implications of key findings; systematic review registration number.	2
**INTRODUCTION**			
Rationale	3	Describe the rationale for the review in the context of what is already known.	4
Objectives	4	Provide an explicit statement of questions being addressed with reference to participants, interventions, comparisons, outcomes, and study design (PICOS).	4
**METHODS**			
Protocol and registration	5	Indicate if a review protocol exists, if and where it can be accessed (e.g., Web address), and, if available, provide registration information including registration number.	4
Eligibility criteria	6	Specify study characteristics (e.g., PICOS, length of follow-up) and report characteristics (e.g., years considered, language, publication status) used as criteria for eligibility, giving rationale.	5
Information sources	7	Describe all information sources (e.g., databases with dates of coverage, contact with study authors to identify additional studies) in the search and date last searched.	5
Search	8	Present full electronic search strategy for at least one database, including any limits used, such that it could be repeated.	Appendix 1
Study selection	9	State the process for selecting studies (i.e., screening, eligibility, included in systematic review, and, if applicable, included in the meta-analysis).	5
Data collection process	10	Describe method of data extraction from reports (e.g., piloted forms, independently, in duplicate) and any processes for obtaining and confirming data from investigators.	5/6
Data items	11	List and define all variables for which data were sought (e.g., PICOS, funding sources) and any assumptions and simplifications made.	Table 1 and 3
Risk of bias in individual studies	12	Describe methods used for assessing risk of bias of individual studies (including specification of whether this was done at the study or outcome level), and how this information is to be used in any data synthesis.	5
Summary measures	13	State the principal summary measures (e.g., risk ratio, difference in means).	5/6
Synthesis of results	14	Describe the methods of handling data and combining results of studies, if done, including measures of consistency (e.g., I^2) for each meta-analysis.	5

For peer review only

PRISMA 2009 Checklist

Section/topic	#	Checklist item	Reported on page #
Risk of bias across studies	15	Specify any assessment of risk of bias that may affect the cumulative evidence (e.g., publication bias, selective reporting within studies).	5/6
Additional analyses	16	Describe methods of additional analyses (e.g., sensitivity or subgroup analyses, meta-regression), if done, indicating which were pre-specified.	N/A
**RESULTS**			
Study selection	17	Give numbers of studies screened, assessed for eligibility, and included in the review, with reasons for exclusions at each stage, ideally with a flow diagram.	6 and Fig 1
Study characteristics	18	For each study, present characteristics for which data were extracted (e.g., study size, PICOS, follow-up period) and provide the citations.	Table 1 and 2
Risk of bias within studies	19	Present data on risk of bias of each study and, if available, any outcome level assessment (see item 12).	Table 2
Results of individual studies	20	For all outcomes considered (benefits or harms), present, for each study: (a) simple summary data for each intervention group (b) effect estimates and confidence intervals, ideally with a forest plot.	Table 1 and 3
Synthesis of results	21	Present results of each meta-analysis done, including confidence intervals and measures of consistency.	N/A
Risk of bias across studies	22	Present results of any assessment of risk of bias across studies (see Item 15).	N/A
Additional analysis	23	Give results of additional analyses, if done (e.g., sensitivity or subgroup analyses, meta-regression [see Item 16]).	N/A
**DISCUSSION**			
Summary of evidence	24	Summarize the main findings including the strength of evidence for each main outcome; consider their relevance to key groups (e.g., healthcare providers, users, and policy makers).	12/13
Limitations	25	Discuss limitations at study and outcome level (e.g., risk of bias), and at review-level (e.g., incomplete retrieval of identified research, reporting bias).	13
Conclusions	26	Provide a general interpretation of the results in the context of other evidence, and implications for future research.	13/14
**FUNDING**			
Funding	27	Describe sources of funding for the systematic review and other support (e.g., supply of data); role of funders for the systematic review.	14/15

From: Moher D, Liberati A, Tetzlaff J, Altman DG, The PRISMA Group (2009). Preferred Reporting Items for Systematic Reviews and Meta-Analyses: The PRISMA Statement. PLoS Med 6(6): e1000097. doi:10.1371/journal.pmed1000097

For more information, visit: www.prisma-statement.org.

For peer review only

For peer review only - http://bmjopen.bmj.com/site/about/guidelines.xhtml

BMJ Open

Views of commissioners, managers and healthcare professionals on the NHS Health Check programme: a systematic review

Journal:	*BMJ Open*
Manuscript ID	bmjopen-2017-018606.R1
Article Type:	Research
Date Submitted by the Author:	15-Sep-2017
Complete List of Authors:	Mills, Katie; University of Cambridge, Public Health & Primary Care<br>Harte, Emma; RAND Europe<br>Martin, Adam; Academic Unit of Health Economics, Leeds Institute of Health Sciences<br>MacLure, Calum; RAND Europe<br>Griffin, Simon; The Primary Care Unit, Institute of Public Health<br>Mant, Jonathan; University of Cambridge, General Practice and Primary Care Research Unit<br>Meads, Catherine; Anglia Ruskin University, Faculty of Health, Social Care and Education<br>Saunders, Catherine; University of Cambridge, Cambridge Centre for Health Services Research<br>Walter, Fiona; University of Cambridge, Dept of Public Health and Primary Care<br>Usher-Smith, Juliet; The Primary Care Unit, Institute of Public Health
**Primary Subject Heading**:	Public health
Secondary Subject Heading:	Cardiovascular medicine, General practice / Family practice, Health policy, Qualitative research
Keywords:	NHS Health Check, health care professional views, systematic review

SCHOLARONE™
Manuscripts

Views of commissioners, managers and healthcare professionals on the NHS Health Check programme: a systematic review

Mills K[1], Harte E[2], Martin A[3], MacLure C[2], Griffin SJ[1,4], Mant J[1], Meads C[5], Saunders CL[1], Walter FM[1], Usher-Smith JA[1]

[1]The Primary Care Unit, Institute of Public Health, University of Cambridge, Box 113 Cambridge Biomedical Campus, Cambridge, CB2 0SR, UK

[2]RAND Europe, Westbrook Centre, Milton Road, Cambridge, CB4 1YG, UK

[3]Academic Unit of Health Economics, Leeds Institute of Health Sciences, University of Leeds, Leeds, LS2 9LJ, UK

[4]MRC Epidemiology Unit, University of Cambridge, Institute of Metabolic Science, Cambridge, CB2 0QQ, UK

[5]Faculty of Health, Social Care and Education, Anglia Ruskin University

Correspondence to: Juliet Usher-Smith jau20@medschl.cam.ac.uk

Key words: NHS Health Check, health care professional views, systematic review

Word count: 3987

For peer review only

ABSTRACT

Objective: To synthesize data concerning the views of commissioners, managers and health-care professionals towards the NHS Health Check programme in general and the challenges faced when implementing it in practice.

Design: A systematic review of surveys and interview studies with a descriptive analysis of quantitative data and thematic synthesis of qualitative data.

Data sources: An electronic literature search of Medline, Embase, Health Management Information Consortium (HMIC), Cumulative Index of Nursing and Allied Health Literature (CINAHL), Global Health, PsycInfo, Web of Science, OpenGrey, the Cochrane Library, NHS Evidence, Google Scholar, Google, Clinical Trials.gov and the ISRCTN registry to 09/11/16 with no language restriction and manual screening of reference lists of all included papers.

Inclusion criteria: Primary research reporting views of commissioners, managers or healthcare professionals on the NHS Health Check programme and its implementation in practice.

Results:

Of 18,524 citations, 15 articles met the inclusion criteria. There was evidence from both quantitative and qualitative studies that some commissioners and general practice healthcare professionals were enthusiastic about the programme while others raised concerns around inequality of uptake, the evidence-base and cost-effectiveness. In contrast, those working in pharmacies were all positive about programme benefits, citing opportunities for their business and staff. The main challenges to implementation were: difficulties with IT and computer software; resistance to the programme from some GPs; the impact on workload and staffing; funding; and training needs. Inadequate privacy was also a challenge in pharmacy and community settings, along with difficulty recruiting people eligible for Health Checks, and poor public access to some venues.

Conclusions:

The success of the NHS Health Check Programme relies on engagement by those responsible for its commissioning, management and delivery. Recognising and addressing the challenges identified in this review, in particular the concerns of GPs, is important for the future of the programme.

For peer review only

Strengths and limitations of this study

- This is the first study to systematically synthesize data concerning the views of commissioners, managers and healthcare professionals on the NHS Health Check programme.
- By including quantitative and qualitative data and studies not published in the mainstream medical literature it provides a comprehensive overview.
- However, the included studies were at risk of selection bias with recruitment consistently reported to have been difficult and all included only small sample sizes.
- Participants may also have responded in ways that reflected best practice or views they felt they ought to hold rather than their true views.

For peer review only

INTRODUCTION

Despite improvements in clinical care and reductions in risk factors such as smoking, cardiovascular disease (CVD) remains the leading cause of years of life lost in the UK[1], with nearly 400 people dying each day from CVD across England and Wales[2]. To help reduce this burden of disease the National Institute for Health and Care Excellence[3,4] and World Health Organization[5] recommend incorporating primary prevention initiatives. To address this, in 2009 Public Health England (PHE) introduced the NHS Health Check programme in England. The aim of the programme is to offer to all those between 40 and 74 years of age, with no pre-existing CVD, type 2 diabetes or dementia, an assessment of their risk of developing CVD and diabetes and advice about risk management, including medication, lifestyle advice and referral services.

The NHS Health Checks are held in General Practice (GP) surgeries, pharmacies, and community settings and are delivered by GPs, practice nurses, Health Care Assistants (HCAs), pharmacists and/or pharmacy assistants. Although it has been a mandated public health service since 2013 with clear guidelines on the required elements[6], there is flexibility in how local areas choose to commission the programme with GP surgeries and pharmacies choosing whether to deliver NHS Health Checks. The programme itself has also remained controversial, and its effectiveness has been challenged by both researchers and clinicians[7–9]. The result has been variability in approach to implementation and delivery across the country[10] and varying levels of engagement amongst health care professionals.

As with all individual-level interventions the impact of the NHS Health Check programme depends on those delivering it. This review synthesizes studies describing the views of commissioners, managers and healthcare professionals towards the NHS Health Check programme, and in doing so explores some of the reasons behind this variation and the challenges faced when implementing the programme.

METHODS

We performed a systematic literature review following a study protocol (available at osf.io/amb4z) that followed the PRISMA guidelines[11].

Search strategy

For peer review only

Published studies were identified from the results of an existing literature review conducted by PHE covering the period from 1st January 1996 to 9th November 2016[12]. This was supplemented by a search in Web of Science and OpenGrey over the same time period. We undertook hand searches of the reference lists of all included publications and performed additional online searches for further publications by named authors identified in the search. Searches completed by PHE included the following sources: Medline, PubMed, Embase, Health Management Information Consortium (HMIC), Cumulative Index of Nursing and Allied Health Literature (CINAHL), Global Health, PsycInfo, the Cochrane Library, NHS Evidence, Google Scholar, Google, Clinical Trials.gov and the ISRCTN registry. Full details of all the search strategies are shown in Appendix 1. No language restrictions were applied.

Study eligibility criteria

Study selection was a two-part process. Initially, studies were screened by title and abstract for potential relevance to the NHS Health Checks. We excluded commentaries, editorials and opinion papers. In the second stage we identified studies reporting the views and experiences of healthcare professionals on NHS Health Checks. Two researchers (JUS and AM) read the full-text of all the potentially relevant studies. Studies for which it was unclear whether or not these inclusion criteria were met were discussed at consensus meetings with the wider research team.

Data extraction, quality assessment and synthesis

Data extraction was completed independently by two researchers (JUS + AM/CS/KM for the quantitative data and JUS + EH/CMa/KM for the qualitative data). Data extracted included study design, time period, recruitment method, participants and analytic method. Studies were also assessed for quality using the Critical Appraisal Skills Programme (CASP) checklist[13] for qualitative studies or a combined CASP checklist for cohort or randomized-controlled trials for the quantitative studies. No studies were excluded on the basis of quality alone.

We synthesized the qualitative data using thematic synthesis approaches which have been described in detail elsewhere[14]. Briefly, after initial reading and re-reading the papers we first coded all findings under the headings of “results” and findings” within the primary studies. We then organised these codes into descriptive, and subsequently analytical, themes. The initial coding was completed by two researchers (JUS and EH/CMa). Each researcher had

For peer review only

experience of conducting and analysing qualitative data and brought their own professional background (academic general practice, public services, health systems and innovation) to the interpretation of the findings. Consensus meetings were held with the wider research team, which included researchers with both clinical and non-clinical backgrounds and those with relevant topic expertise, to discuss the emerging codes and develop descriptive and analytical themes. To allow for appreciation of the data reviewed in these studies, illustrative quotations have been included alongside the analytical themes presented.

For the quantitative data, we extracted all the findings from the studies and synthesized those descriptively, grouping similar aspects together.

RESULTS

The initial literature search generated 18,524 titles and abstracts. 178 papers were potentially relevant to NHS Health Checks. These were reviewed at full text level (Figure 1). Of those, 164 were excluded. Reasons were that they did not include any relevant data for this research question, were duplicates or commentaries, or did not describe NHS Health Checks. Through citation searching one additional article was identified. This review is, therefore, based on 15 articles[15–29]. The characteristics of these are shown in Table 1 and the detailed quality assessment is shown in Tables 2a and b.

The studies used a range of designs. One included quantitative results from surveys[19], two quantitative results from surveys alongside free-text question responses[15,18,22], one free-text responses from a survey[29], and 10 findings from semi-structured interviews[16,17,20,21,23–28]. The majority (*n*=10) reported the views of healthcare professionals working within general practice. Four included pharmacists[17,19,21,23], four those delivering NHS Health Checks within community settings[23,25,26,29], and two commissioners[21,23]. Most collected data within the first two years of the programme (2009-2011). Sample sizes ranged between 25 and 442 for the survey studies and between 4 and 58 for the interview studies. All of the qualitative studies were considered to be of medium or high quality and the three quantitative studies were all of medium quality. Response rates for the two survey studies that reported them were 24% for GPs[18], 76% for practice managers[18], and 34% for pharmacists[19].

Overall views of the NHS Health Check Programme

For peer review only

Commissioners

Only one study reported the views of commissioners on the programmes as a whole[23]. Across the 14 commissioners interviewed, their enthusiasm for NHS Health Checks varied: while many approaching the programme positively, others described lower levels of engagement.

> "*It's very difficult to provide reassurance when on a personal level you're not sure if you've 100% bought into the programme either*" Commissioner[23]

General practice healthcare professionals

Two studies reported quantitative results from surveys with general practice healthcare professionals. In one, a survey of 43 GPs from 31 practices[18], 51% (*n*=22) viewed the programme as important, 54% (*n*=24) as beneficial to their patients, and 5% (*n*=2) considered the NHS Health Check programme to be a waste of time and resources. In the same study 36 out of 81 GPs and practice managers (44%) felt the high risk patient identification was beneficial to the practice. In a second survey of 25 healthcare professionals 72% (*n*=18) perceived that NHS Health Checks were useful in early detection and gave time to discuss patient health and lifestyles[15].

Of the ten interview studies, in general participants expressed the view that NHS Health Checks were beneficial in the early detection and prevention of disease[16,20,23,27].

> *"It's a good way to try and prevent illness and long term or serious conditions developing in the future"* Practice manager[20]

> *"I think it's a very good idea. We have a very high proportion of our patients who suffer with diabetes, almost 10% of our patients are diabetic so I thought this was an excellent opportunity to screen those earlier and pick them up."* GP[27]

There were, however, a number of concerns raised about the programme. In particular, some GPs described how they felt the programme attracted the "*worried well*" and that the patients who would benefit the most were the ones who were least likely to attend[15,16,22–24].

For peer review only - http://bmjopen.bmj.com/site/about/guidelines.xhtml

> *"if you send out an invite to a large number of people then the people who present themselves (laughs) er might well fit into that worried well category, um won't necessarily be um the HGV driver who works long hours and smokes a lot"* GP[24]

Many also described doubts about the long-term benefits and the costs of implementation, including staff resources and lack of evidence for the effectiveness[15,16,18,20,24].

> *"I don't think there is an awful lot of value. I think you'll pick up a few people a little bit earlier. Now whether that's worth the cost, obviously it's great for those individual patients, whether that's worth the cost of running a programme like this. I'd be amazed if it was."* Nurse[24]

> *"I think really this is mass screening and there's not a great deal of proof behind it…..Not entirely convinced with being told we have to offer a check to everyone."* GP[16]

Linked to this, participants in several of the studies described the challenges to achieving behaviour change and the difficulties they had getting people to make longstanding changes to their lifestyle following the health checks[27] [16,26] [15].

> *"Even if you access them, even if you find out that they're a really high risk score then getting these people to take on board you know the lifestyle changes, changes to their diet, exercising more. It's very difficult to get them to take those changes on."* Nurse[16]

Managing high-risk levels of alcohol consumption was felt to be especially challenging for some GPs and staff, particularly amongst patients in certain religious groups in which alcohol consumption can be stigmatised[27]. A lack of resources and lack of, or inconsistency of, well-funded support services in the wider community also contributed to this[15,16,23,27].

> *"We used to have things called exercise referral and we refer people to free gym sessions and send them to Slimming World and they'd get Slimming World sessions. We had really good responses and really good uptake for that, but that's all gone now."* Nurse[16]

For peer review only

Pharmacists

Three studies described the views of pharmacists[17,19,21]. Two of these are conference abstracts in which pharmacists and those involved in the delivery of NHS Health Checks in pharmacies had been interviewed[17,19]. The third sent out a postal questionnaire to pharmacists, reporting a 34% response rate[21]. In contrast to the studies with healthcare professionals from general practice, very few participants from pharmacies discussed the benefits or otherwise of the NHS Health Checks to patients. Instead the focus was on the benefits of delivering NHS Health Checks in pharmacies, with all feeling it offered immense job satisfaction, promoted the image of the pharmacy and provided a good opportunity for staff development[17,19,21].

> "*I wanted to do this regardless... if I'm in a position where I can give somebody information that will then enable them to change their behaviour and live a healthier life that's a satisfying thing to do*." Pharmacist[17]

> *"For being the place to come in your local area for your health concerns, I think all round, for both the staff personally and for the company's goal, I think it's a positive thing."* Pharmacist[17]

Those delivering NHS Health checks in community settings

No studies reported the views towards the programme as a whole from those involved in delivering NHS Health Checks in community settings.

Challenges to implementation

One study reported challenges to implementation across all settings reported by commissioners[23]. In that study the greatest challenges were: engaging with GPs, both to encourage them to deliver NHS Health Checks within their practice and to facilitate delivery by non-GP providers; difficulties with data management in the absence of standard Read Codes when NHS Health Checks were first introduced, and the lack of clear national guidelines around data handling; and ensuring consistency of provision across GPs, particularly with the lack of a formal quality assurance or monitoring system at the time.

> *"The massive thing is the sheer variability in delivery. You get some star performers and some people that just won't engage with it"* Commissioner[23]

For peer review only

General practice

Seven studies described the challenges general practice healthcare professionals had experienced when implementing the NHS Health Checks within their practice. The main challenges are summarised in Table 3. Difficulties with IT and computer software were mentioned in over half of the studies, particularly related to the call and recall system when the programme was introduced[16,18,22,23] with 39% of practice managers in one study reporting difficulties with the clinical system, software, or errors in the existing data[18]. Impact on workload was also cited as a challenge for some. In a survey of 25 healthcare professionals, approximately 40% indicated there had been issues with staffing levels since starting to deliver NHS Health Checks, with some attributing these issues to the extra workload created by NHS Health Checks[15].

> *"NHS Health Check generates a huge workload for our staff in addition to what we do, a roughly 20 per cent additional workload*" Nurse[15]

In other studies, practice managers and GPs also generally agreed that the programme's impact on workload had knock-on effects on other services[18], with the financial reimbursement considered not sufficient to justify the work[18,23,27] or influencing their implementation[27].

> *"In order to get good payments we had to reach 50% target within three months …it was important for us to get the targets very very quickly*.' GP[27]

Concerns about remuneration were also reported by commissioners who claimed that NHS Health Checks were less of a priority as they are not part of the Quality and Outcomes Framework (QOF) for which GPs get paid[23].

> *"GPs have a very 'small business' mentality, and if the Health Care Assistant is off doing a Health Check and can't be doing something else for them, they get very jittery about that"* Commissioner[23]

Inadequate training was the final theme and was discussed in many of the studies[15,16,22,27]. These include a survey of 25 health care professionals in which 44% (*n*=11) indicated that

For peer review only

they required further training[15]. A survey of staff at 65 general practices in two inner London boroughs showed that staff at 62% (*n*=40) and 65% (*n*=42) of practices had attended training in delivering lifestyle advice or risk information, but only 43% (*n*=28) of practices reported that staff had attended training in measurement methods; at 23% (*n*=15) of practices no specific training was reported and 28% (*n*=18) considered that additional training would have been beneficial[22]. In free text responses 24% (*n*=5/21) of health care professionals suggested that improvements to staff training and capacity were required [22].

> *"[Training] would be good. As I say, we just learnt from our healthcare assistant what to do; basically it was like kind of on the job training… It would be nice to understand it in depth more, wouldn't it?"* HCA [16]

Pharmacies

Three studies, two of which are conference abstracts, reported the challenges faced by those involved in commissioning or delivering NHS Health Checks within pharmacies. In a survey of 442 community pharmacists[19], the three most important perceived barriers to implementation were lack of time, lack of staff and lack of reimbursement (all reported by over 55% of respondents). Lack of time and staff were also referred to in qualitative interviews with pharmacy staff. In particular, they described how, due to other commitments, most pharmacists did not have the capacity to perform the initial assessments as part of the NHS Health Checks. Instead, these were carried out by pharmacy assistants, who in turn needed more substantial training than was initially offered[17,19,21]

> "*The people they have working for them… haven't got the background in care knowledge or expertise. It wasn't like a GP surgery where you have Healthcare Assistants and Practice Nurses who on a day to day basis take blood pressures, take pulses, take blood and give advice on health*" PCT staff member[21]

Difficulties with funding were also discussed by commissioners who had had to develop different agreements from those with general practice as pharmacies pay VAT on all services they deliver and had had to allocate additional funds for unexpected costs, such as having to vaccinate pharmacy staff to allow them to handle blood and bodily fluids[21].

For peer review only

Other challenges (Table 3) identified by pharmacists and commissioners included: lack of private space for consultations (25% (*n*=111/442) [19]); difficulties with IT, particularly the need for a sufficiently secure internet connection to allow them to transfer patient identifiable data; and difficulty recruiting participants as the eligible population was largely dictated by footfall within the pharmacy[19,21,23]. Some pharmacies that were very close to GP practices delivering the NHS Health Check, also experienced competition between settings.

> *"Actually there's another problem, capturing the people. Everyone is out to capture them...it's very hard if you see someone coming in and say, 'Oh! You could be a candidate', and they say, 'The surgery has approached me and I'm going there'."* Pharmacy representative[21]

Community settings

Three studies reported the views of those involved in delivering NHS Health Checks in community settings[25,2923]. In contrast to some of the views expressed by HCAs working in general practice, in a small study of 10 HCAs delivering community-based NHS Health Checks, most felt there were enough staff and felt they had adequate support[29] and workers on a Health Bus found delivering NHS Health Checks to be fulfilling, enjoyable and overall a positive experience[23]. The main challenges identified (Table 3) were poor access to some venues, inadequate privacy, problems with some of the equipment and connection to the internet, and resistance from GPs to accept referrals from third-party providers.

> *"I don't think you come across very professional when you're sitting in a kitchen and all huddled round and all on top of each other. And it's not very nice for the patients, because…quite personal information"* Nurse[25]

> *"Because we were all in the same room it was easy to listen to what was happening next door."* HCA[29]

DISCUSSION

Key findings

While there was evidence that some commissioners, managers and healthcare professionals working in general practice could see the benefit of the NHS Health Check programme for patients, in the largest survey of GPs only half viewed the programme as important and

beneficial to their patients. A range of views was also seen in qualitative studies where some were enthusiastic while others raised concerns around inequality of uptake, the evidence behind the programme, and the cost-effectiveness. In contrast, those working in pharmacies were all positive about the programme, citing opportunities for their business and staff as reasons.

A number of challenges to implementation were identified. Difficulties with IT and computer software and resistance to the programme from GPs were described across all settings. The impact on workload and staffing, funding, and training needs were also challenges in general practice and pharmacy settings, while inadequate privacy was common to both pharmacies and community settings. Some pharmacies also experienced difficulty recruiting people for NHS Health Checks and poor access to some venues was reported in community settings.

Strengths and limitations

The strengths of this review include the comprehensive electronic search across multiple databases, the inclusion of reports not published within the mainstream medical literature, and the synthesis of both quantitative and qualitative data. However, all the studies included only small sample sizes and so may not be generalizable beyond the study context. In addition, recruiting GPs was consistently reported to have been difficult, especially from practices performing fewer NHS Health Checks, and the pharmacists who took part were all from pharmacies already involved in delivering NHS Health Checks; these studies are therefore at a particular risk of selection bias. Although the studies included a range of professionals from different settings, the views reported may, therefore, reflect the opinions of those who are particularly enthusiastic or negative, or have strong views about the NHS Health Check programme. The findings are also constrained by the questions addressed by the original researchers. Secondly, across all the studies it is possible that participants responded in ways that reflected best practice or the views they felt they ought to hold and so the findings may not reflect their true personal views. We also did not have access to the original data and so were only able to synthesize the findings considered by the authors of the original studies as worthy of report. Finally, all but two studies were conducted prior to 2013 and so are more representative of the initial phase of the programme and may not reflect changes since then.

Comparison with existing literature

For peer review only

While we only included studies specific to the NHS Health Check programme, the main challenges to implementation identified in this study are consistent with those reported for prevention and health promotion in general. A multinational study across 11 European countries which included over 2000 GPs found that, although GPs believed prevention and health promotion was important, the workload, lack of time and need for funding limited their engagement[30]. Issues around workload and lack of time were also the two main barriers in a survey of GP views of their role in cancer prevention in the UK[31] and, along with lack of funding, were reported in a questionnaire survey of general practice healthcare professionals views on advising patients about physical activity[32] and a qualitative study of lifestyle counselling in Ireland[33]. The concerns expressed by some healthcare professionals in this study about the difficulties changing patients' behaviours are also commonly reported in the literature[32,34–36]: in one survey 40.3% (n = 112/278) GPs agreed that patients' behaviours are established and difficult to change[31].

Implications for clinicians, policymakers and future research

Given their central role in the success of the programme, the finding that a number of commissioners, GPs and other general practice staff had doubts about the evidence behind the programme has important implications for future delivery of NHS Health Checks. Lack of belief about proven effectiveness has been identified as one of the main barriers to offering health promotion activities within routine care amongst Dutch GPs and nurses[36] and evidence of effectiveness as one of the main incentives for GPs in a multinational study[30]. A survey of Australian GPs' views on clinical guidelines also cited an evidence base as the most important factor in their deciding whether to follow the recommendations of a guideline[37]. In the eight years since the programme was introduced there has been a growing evidence base around the NHS Health Checks. In contrast to the views held by many of the healthcare professionals in these studies, evidence suggests, for example, that, in part due to targeted approaches, more people in the most deprived quintile compared with the least deprived quintile have had NHS Health Checks[38–40] and there has been a consistent 3% to 4% increase in statin prescribing amongst attendees of the NHS Health Check compared with matched non-attendees[41–43]. Ensuring that programmes are effective and producing up-to-date, concise, summaries of the evidence and estimated benefits for different patient groups in an easily accessible format should therefore be a priority for those supporting delivery of the NHS Health Check programme and other similar prevention programmes. Piloting future programmes to provide such evidence before rolling them out nationally and including a

phased roll out with in-built evaluation may also help address some of these concerns, particularly amongst GPs whose engagement is key to delivery of the programme in all settings.

Anticipating and addressing training needs and difficulties with IT and computer software early may also increase engagement. Indeed, in 2013, since the majority of these studies were published, PHE introduced standard Read codes to facilitate data entry, updated software for identifying those eligible, and provided additional online training modules for healthcare professionals.

Overcoming some of the other challenges identified, such as funding and increased workload, are more difficult given the context of the current financial crisis within the NHS and reports of primary care services being stretched beyond safe limits by the needs of those with existing morbidity[44]. However, this review suggests there may be greater enthusiasm amongst pharmacies than general practices for delivering NHS Health Checks. Capitalising on this may be an effective way to reduce pressure on general practice whilst at the time empowering pharmacists to take on a wider role within healthcare[45].

ACKNOWLEDGEMENTS

We thank our patient and public representatives Kathryn Lawrence and Chris Robertson for providing helpful comments on the findings and the NHS Health Checks Expert Scientific and Clinical Advisory Panel working group for providing us with the initial literature search conducted by Public Health England. We would also like to thank Anna Knack, Research Assistant at RAND Europe, for her excellent research support, and Emma Pitchforth for her helpful comments on our analysis.

A summary of the findings reported in this manuscript have been published online by Public Health England (available at http://www.healthcheck.nhs.uk/commissioners_and_providers/evidence/) and RAND (http://www.rand.org/content/dam/rand/pubs/external_publications/EP60000/EP67129/RAND_EP67129.pdf). Permission from both has been obtained to publish the results in this journal.

Contributors

KM synthesised and interpreted the findings and wrote the first draft of the manuscript. EH screened articles for inclusion, extracted and synthesised the qualitative data, interpreted the findings and critically revised the manuscript. CMa extracted and synthesised the qualitative data and critically revised the manuscript. AM screened articles for inclusion, interpreted the findings and critically revised the manuscript. CS, CM, FW, SG and JM developed the protocol, interpreted the findings and critically revised the manuscript. JUS developed the protocol, screened articles for inclusion, extracted and synthesised the quantitative and qualitative data, interpreted the findings and wrote the first draft of the manuscript.

Funding

This work was funded by a grant from Public Health England. JUS and KM are funded by a Cancer Research UK/BUPA Foundation Cancer Prevention Fellowship (C55650/A21464) and FW by an NIHR Clinician Scientist award. The views expressed in this publication are those of the authors and not necessarily those of the NHS, the NIHR or the Department of Health.

All researchers were independent of the funding body and the funder had no role in data collection, analysis and interpretation of data; in the writing of the report; or decision to submit the article for publication.

Data sharing

All data are available from the reports or authors of the primary research. No additional data is available.

Competing Interests

All authors have completed the Unified Competing Interest form at *www.icmje.org/coi_disclosure.pdf* (available on request from the corresponding author) and declare that (1) they have no support from or relationships with companies that might have an interest in the submitted work in the previous 3 years; (2) their spouses, partners, or children have no financial relationships that may be relevant to the submitted work; and (3) they have no non-financial interests that may be relevant to the submitted work.

The corresponding author has the right to grant on behalf of all authors and does grant on behalf of all authors, an exclusive licence on a worldwide basis to the BMJ Publishing Group

For peer review only

Ltd and its Licensees to permit this article (if accepted) to be published in BMJ editions and any other BMJPGL products and sub-licences to exploit all subsidiary rights, as set out in our licence (http://resources.bmj.com/bmj/authors/checklists-forms/licence-for-publication).

All authors had full access to all of the data in the study and can take responsibility for the integrity of the data and the accuracy of the data analysis

The corresponding author affirms that the manuscript is an honest, accurate, and transparent account of the study being reported; that no important aspects of the study have been omitted; and that any discrepancies from the study as planned (and, if relevant, registered) have been explained.

FIGURE LEGENDS

Figure 1. PRISMA flow diagram

REFERENCES

1 Murray CJL, Richards MA, Newton JN, *et al.* UK health performance: findings of the Global Burden of Disease Study 2010. *Lancet (London, England)* 2013;**381**:997–1020.

2 Office for National Statistics Death Registrations. 2015.https://www.ons.gov.uk/peoplepopulationandcommunity/birthsdeathsandmarriages/deaths/datasets/deathregistrationssummarytablesenglandandwalesreferencetables (accessed 19 May2017).

3 National Institute for Health and Care Excellence. Cardiovascular disease prevention Public health guideline [PH25]. 2010.https://www.nice.org.uk/guidance/ph25 (accessed 19 May2017).

4 National Institute for Health and Care Excellence. Cardiovascular disease: risk assessment and reduction, including lipid modification Clinical guideline [CG181]. 2016.https://www.nice.org.uk/guidance/cg181 (accessed 19 May2017).

5 World Health Organisation. Package of Essential Noncommunicable interventions for primary health care in low-resource settings. www.who.int/nmh/publications/essential_ncd_interventions_lr_settings.pdf (accessed 19 May2017).

6 Public Health England. NHS Health Check Best practice guidance. Published Online First: 2015.www.healthcheck.nhs.uk/document.php?o=456

7 Capewell S, McCartney M, Holland W. Invited debate: NHS Health Checks-a naked emperor? *J Public Health (Oxf)* 2015;**37**:187–92.

8 Krogsbøll LT, Jørgensen KJ, Gøtzsche PC. General health checks in adults for reducing morbidity and mortality from disease. *JAMA* 2013;**309**:2489–90.

9 Krogsbøll LT, Jørgensen KJ, Grønhøj Larsen C, *et al.* General health checks in adults for reducing morbidity and mortality from disease: Cochrane systematic review and

meta-analysis. *BMJ* 2012;**345**:e7191.

10 Graley CEM, May KF, McCoy DC. Postcode lotteries in public health--the NHS Health Checks Programme in North West London. *BMC Public Health* 2011;**11**:738.

11 Moher D, Liberati A, Tetzlaff J, *et al.* Preferred reporting items for systematic reviews and meta-analyses: the PRISMA statement. *Int J Surg* 2010;**8**:336–41.

12 Public Health England. NHS Health Check literature review. http://www.healthcheck.nhs.uk/commissioners_and_providers/evidence/literature_review/

13 Critical Appraisal Skills Programme (CASP) Checklists. http://www.casp-uk.net/casp-tools-checklists

14 Thomas J, Harden A. Methods for the thematic synthesis of qualitative research in systematic reviews. *BMC Med Res Methodol* 2008;**8**:45.

15 Baker C, Loughren EA, Crone D, *et al.* Perceptions of health professionals involved in a NHS Health Check care pathway. *Pract Nurs* 2015;**26**:608–12.

16 Ismail H, Kelly S. Lessons learned from England's Health Checks Programme: using qualitative research to identify and share best practice. *BMC Fam Pract* 2015;**16**:144.

17 Crabtree V, Hall J, Gandecha M. NHS health checks: The views of community pharmacists and support staff. *Int J Pharm Pract* 2010;**18**:35–6.

18 Krska J, du Plessis R, Chellaswamy H. Views of practice managers and general practitioners on implementing NHS Health Checks. *Prim Health Care Res Dev* 2015;:1–8.

19 Loo RL, Diaper C, Salami OT, *et al.* The NHS Health Check: The views of community pharmacists. *Int J Pharm Pract* 2011;**19**:13.

20 McDermott L, Wright AJ, Cornelius V, *et al.* Enhanced invitation methods and uptake of health checks in primary care: randomised controlled trial and cohort study using electronic health records. *Health Technol Assess (Rockv)* 2016;**20**:1–92.

21 McNaughton RJ, Oswald NT, Shucksmith JS, *et al.* Making a success of providing NHS Health Checks in community pharmacies across the Tees Valley: a qualitative study. *BMC Health Serv Res* 2011;**11**:1–7.

22 Nicholas JM, Burgess C, Dodhia H, *et al.* Variations in the organization and delivery of the 'NHS health check' in primary care. *J Public Heal (United Kingdom)* 2013;**35**:85–91.

23 Research Works. Public Health England Understanding the implementation of NHS Health Checks. 2013.

24 Riley R, Coghill N, Montgomery A, *et al.* Experiences of patients and healthcare professionals of NHS cardiovascular health checks: A qualitative study. *J Public Health (Bangkok)* 2015;**38**:543–51.

25 Riley R, Coghill N, Montgomery A, *et al.* The provision of NHS health checks in a community setting: an ethnographic account. *BMC Health Serv Res* 2015;**15**:1–10.

26 Shaw RL, Pattison HM, Holland C, *et al.* Be SMART: examining the experience of implementing the NHS Health Check in UK primary care. *BMC Fam Pract* 2015;**16**:1.

27 Shaw RL, Lowe H, Holland C, *et al.* GPs' perspectives on managing the NHS Health Check in primary care: A qualitative evaluation of implementation in one area of England. *BMJ Open* 2016;**6**:e010951.

28 Oswald N, Mcnaughton R, Watson P, *et al.* Tees Vascular Assessment Programme

Evaluation. 2010.

29 NHS Greenwich. Evaluation of NHS Health Check PLUS Community Outreach Programme in Greenwich. 2011;:1–61.

30 Brotons C, Björkelund C, Bulc M, *et al.* Prevention and health promotion in clinical practice: the views of general practitioners in Europe. *Prev Med (Baltim)* 2005;**40**:595–601.

31 McIlfatrick S, Keeney S, McKenna H, *et al.* Investigating the role of the general practitioner in cancer prevention: a mixed methods study. *BMC Fam Pr* 2013;**14**:58.

32 Douglas F, Torrance N, van Teijlingen E, *et al.* Primary care staff's views and experiences related to routinely advising patients about physical activity. A questionnaire survey. *BMC Public Health* 2006;**6**:138.

33 Lambe B, Collins C. A qualitative study of lifestyle counselling in general practice in Ireland. *Fam Pract* 2010;**27**:219–23.

34 Jallinoja P, Absetz P, Kuronen R, *et al.* The dilemma of patient responsibility for lifestyle change: perceptions among primary care physicians and nurses. *Scand J Prim Health Care* 2007;**25**:244–9.

35 Jacobsen ET, Rasmussen SR, Christensen M, *et al.* Perspectives on lifestyle intervention: the views of general practitioners who have taken part in a health promotion study. *Scand J Public Health* 2005;**33**:4–10.

36 Geense WW, van de Glind IM, Visscher TLS, *et al.* Barriers, facilitators and attitudes influencing health promotion activities in general practice: an explorative pilot study. *BMC Fam Pract* 2013;**14**:20.

37 Gupta L, Ward JE, Hayward RS. Clinical practice guidelines in general practice: a national survey of recall, attitudes and impact. *Med J Aust* 1997;**166**:69–72.

38 Forster AS, Dodhia H, Booth H, *et al.* Estimating the yield of NHS Health Checks in England: A population-based cohort study. *J Public Heal (United Kingdom)* 2015;**37**:234–40.

39 Chang K-M, Soljak M, Lee JT, *et al.* Coverage of a national cardiovascular risk assessment and management programme (NHS Health Check): Retrospective database study. *Prev Med (Baltim)* 2015;**78**:1–8.

40 Robson J, Dostal I, Sheikh A, *et al.* The NHS Health Check in England: an evaluation of the first 4 years. *BMJ Open* 2016;**6**:e008840.

41 Chang K, Lee J, Vamos E, *et al.* Impact of the National Health Service Health Check. *CMAJ* 2016;**188**:E228-238.

42 Forster AS, Burgess C, Dodhia H, *et al.* Do health checks improve risk factor detection in primary care? Matched cohort study using electronic health records. *J Public Health (Bangkok)* 2015;:1–8.

43 Robson J, Dostal I, Madurasinghe V, *et al.* NHS Health Check comorbidity and management : an observational matched study in primary care. *Br J Gen Pract* Published Online First: 2016. doi:10.3399/bjgp16X688837

44 The Kings Fund. Understanding pressures in general practice. 2016.http://www.kingsfund.org.uk/sites/files/kf/field/field_publication_file/Understanding-GP-pressures-Kings-Fund-May-2016.pdf

45 Royal Pharmaceutical society. Now or never: Shaping pharmacy for the future. 2013.https://www.rpharms.com/resources/reports/now-or-never-shaping-pharmacy-

For peer review only - http://bmjopen.bmj.com/site/about/guidelines.xhtml

for-the-future

For peer review only

Table 1. Features of studies

Author/ year	Type of report	Study period	Location of study	Setting of NHS Health Check	Data collection method	*n*	Method of recruitment to study	Participant characteristics	Method of analysis
Baker 2015[15]	Journal article	Not given	South West England	30.1% of total practices delivering NHS Health Checks	Surveys including quantitative and qualitative questions	25	Identified randomly via the County Medical List to ensure geographic spread	2 GPs, 14 practice managers, 6 practice nurses, 2 healthcare assistants and 1 administrator	Descriptive statistics Thematic analysis
Crabtree 2010[17]	Conference abstract	2009	Not given	32 (of 35) pharmacies in the area delivering NHS Health Checks	Semi-structured telephone interviews	32	All 35 pharmacies delivering the service were contacted	15 pharmacists, 13 support staff and 4 pre-registration pharmacists	Thematic analysis
Greenwich 2011[29]	Report	2011	Greenwich	Community	Open-ended questionnaire	11	All (12) clinicians delivering community outreach services providing NHS Health Checks were invited	Healthcare assistants, nurses, pharmacists and health trainers	Not described
Ismail and Kelly 2015[16]	Journal article	2010	Yorkshire	25 general practices	Semi-structured interviews	58	Letters of invitation or flyers to 41 general practices targeted to reflect diversity in terms of performance	Healthcare assistants, GPs, practice managers, practice nurses and other support staff	Framework analysis
Krska 2015[18]	Journal article	2011	Sefton, an area of North West England	33 (of 55) general practices	Postal survey with free text responses	83 (76% of practice managers, 24% of GPs)	Personally addressed letters of invitation with a covering letter to all practice managers and GPs at 55 practices	40 practice managers and 43 GPs	Categorisation of responses
Loo 2011[19]	Conference abstract	2009	Not given	Pharmacies	Postal questionnaire	442 (34%)	Questionnaire posted to all pharmacies in the area	All pharmacists 59% male; 89.1% full time; 53.4% worked for large multiple pharmacies	Descriptive statistics
McDermott 2016[20]	Journal article	2013-15	2 London boroughs	17 general practices	Semi-structured interviews	24	Recruited from within a trial of an enhanced invitation method	52% practice managers, 9% healthcare assistants, 30% administrators, 9% public health leads	Framework analysis
McNaughton 2011[21]	Journal article	Not given	Tees Valley	8 pharmacies	Semi-structured interviews	20	Postal invitation	10 primary care trust members, 8 pharmacists, 2 representatives from Local Pharmaceutical Committee	Thematic analysis
Nicholas 2012[22]	Journal article	2011	2 London boroughs	70 (of 96) general practices	Survey with free text responses	65	Invitations to all 96 general practices	25 practice managers, 8 GPs, 16 practice nurses, 2 healthcare assistants, 3	Descriptive statistics Content

								administrators and 14 not specified	analysis
Oswald 2010[28]	Evaluation report	2009 – 2010	Teesside	13 general practices	Semi-structured interviews	25	Letter of invitation to practice managers	8 practice managers, 14 practice nurses, 1 GP, 1 healthcare assistant, 1 pharmacist	Thematic analysis
Research works 2013[23]	Research report	2013	Not given	All settings	Semi-structured interviews	26	Contacts provided by Commissioners with snowballing recruitment	14 commissioners, 12 GPs, practice managers, health care assistant, nurse practitioner, physical activity development officer, health bus workers and a community pharmacist	Not described
Riley 2015[25]	Journal article	2013	Bristol inner-city	Community settings	Semi-structured interviews	4	Participants were recruited via their involvement with community outreach events.	1 practice nurse, 1 healthcare assistant, 1 engagement worker and 1 health trainer	Thematic analysis
Riley 2015[24]	Journal article	2013-14	Bristol	11 general practices	Semi-structured interviews	15	18 were invited with purposive sampling	5 GPs, 5 practice nurses, 3 healthcare assistants, 2 pharmacists	Thematic analysis
Shaw 2015[26]	Journal article	2010-11	Birmingham and Black Country	General practices and community	Semi-structured interviews	31	Recruited through lead clinicians	9 GPs, 6 practice managers, 4 practice nurses, 6 healthcare assistants, 1 alternative provider director, 1 call centre manager, 2 call centre operatives and 2 alternative provider registered practice nurses	Thematic analysis
Shaw 2016[27]	Journal article	Not given	Birmingham	General practices	Semi-structured interviews	9	Recruitment undertaken by local NHS trust. No further details provided	All GPs	Thematic analysis

Table 2a. Quality assessment of studies including surveys

Author, date	Study addressed a clearly focused issue	Use of an appropriate method / Randomisation (for RCTs)	Recruitment / comparability of study groups at baseline	Blinding (for RCTs)	Exposure measurement	Outcome measurement	Comparability of study groups during study (for RCTs)	Follow up (for longitudinal studies)	Confounding factors (for non-RCTs):	Applicability to England	Overall
Baker 2015[15]	⬤	●	●	n/a	n/a	●	n/a	n/a	•	•	Medium
Krska 2015[18]	⬤	⬤	●	n/a	n/a	●	n/a	n/a	•	●	Medium
Loo 2011[19]	⬤	⬤	●	n/a	n/a	n/a	n/a	n/a	•	●	Medium

• Low ● Medium ⬤ High

Table 2b. Quality assessment of studies including qualitative data

Author, date	Study addressed a clearly focused issue	Appropriateness of qualitative method	Design	Recruitment	Consideration of relationship between research and participants	Ethical issues	Rigor of data analysis	Clarity of statement of findings	Overall
Baker 2015[15]	⬤	⬤	⬤	•	•	●	●	⬤	Medium
Crabtree 2010[17]	⬤	⬤	•	•	•	●	•	●	Medium
Greenwich 2011[29]	⬤	⬤	⬤	●	•	•	•	●	Medium
Ismail and Kelly 2015[16]	⬤	⬤	⬤	●	●	⬤	⬤	⬤	High
Krska 2015[18]	⬤	⬤	●	⬤	n/a	●	●	●	Medium
McDermott 2016[20]	⬤	⬤	⬤	•	•	●	●	●	Medium
McNaughton 2011[21]	⬤	⬤	⬤	⬤	•	⬤	⬤	⬤	High
Nicholas 2013[22]	⬤	⬤	⬤	⬤	n/a	•	⬤	⬤	High
Oswald 2010[28]	⬤	⬤	⬤	●	•	●	●	●	Medium
Research Works 2013[23]	⬤	⬤	⬤	●	•	•	•	●	Medium
Riley 2015[24]	⬤	⬤	⬤	●	●	⬤	⬤	⬤	High
Riley 2015[25]	⬤	⬤	⬤	●	●	⬤	⬤	⬤	High
Shaw 2015[26]	⬤	⬤	⬤	●	•	⬤	⬤	⬤	High
Shaw 2016[27]	⬤	⬤	⬤	●	•	●	⬤	⬤	High

• Low ● Medium ⬤ High

Table 3. Challenges to implementation of NHS Health Checks reported across the settings

**Challenge to implementation**	**General practices**	**Pharmacies**	**Community settings**
Difficulties with IT and computer software	✓[16,18,22,23]	✓[21,23]	✓[23,25,29]
Impact on workload / staffing	✓[15,16,18,22]	✓[17,19,21]	
Funding	✓[16 18]	✓[19,21]	
Training needs	✓[15,16,22,27]	✓[19,21]	
Resistance from GPs	✓[23]	✓[23]	✓[23]
Inadequate privacy		✓[19,21,23]	✓[25,29]
Difficulty recruiting participants		✓[21,23]	
Poor access to some venues			✓[29]

For peer review only

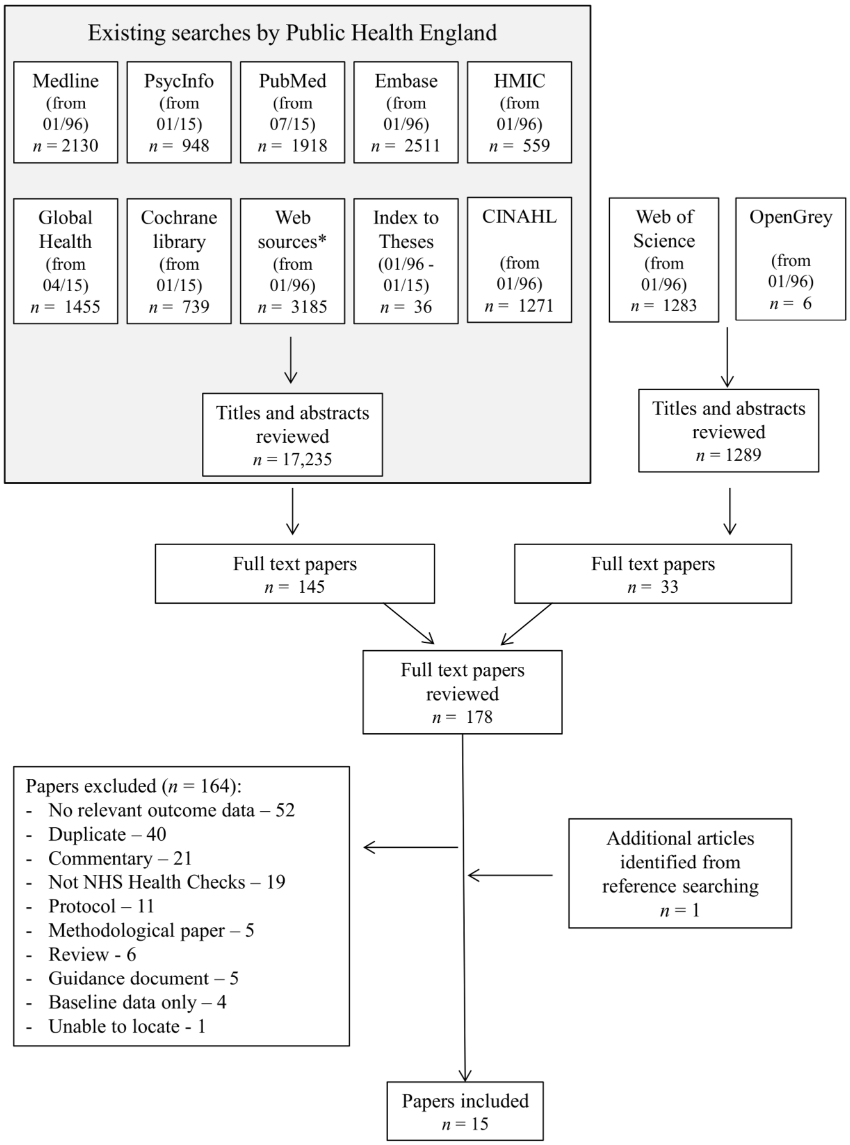

PRISMA flow diagram

190x254mm (300 x 300 DPI)

Appendix 1 – Search strategies

Database	Search strategy
Ovid Medline	1. health check*.tw.<br>2. (diabetes adj3 screen*).tw.<br>3. (cardiovascular adj3 screen*).tw.<br>4. (population adj2 screen*).tw.<br>5. (risk factor adj3 screen*).tw.<br>6. (opportunistic adj3 screen*).tw.<br>7. medical check*.tw.<br>8. general check*.tw.<br>9. periodic health exam*.tw.<br>10. annual exam*.tw.<br>11. annual review*.tw.<br>12. NHSHC.tw.<br>13. 1 or 2 or 3 or 4 or 5 or 6 or 7 or 8 or 9 or 10 or 11 or 12<br>14. cardiovascular adj3 prevention.tw.<br>15. (primary care or general practice or primary healthcare).tw<br>16. 14 and 15<br>17. Cardiovascular Diseases/ AND Primary Prevention/<br>18. 16 or 17<br>19. 13 or 18
PubMed	1. health check*<br>2. diabetes screen*<br>3. cardiovascular screen*<br>4. population screen*<br>5. risk factor screen*<br>6. opportunistic screen*<br>7. medical check*<br>8. general check*<br>9. periodic health exam*<br>10. annual exam*<br>11. annual review*<br>12. NHSHC<br>13. 1 or 2 or 3 or 4 or 5 or 6 or 7 or 8 or 9 or 10 or 11 or 12<br>14. Cardiovascular Diseases AND Primary Prevention[MeSH Terms]<br>15. "primary care"[Text Word] OR "general practice"[Text Word] OR "primary healthcare"[Text Word])<br>16. (cardiovascular[Text Word] AND prevention[Text Word])<br>17. #15 and #16<br>18. #14 or #17<br>19. #13 or #18
Ovid Embase	1. health check*.tw.<br>2. (diabetes adj3 screen*).tw.<br>3. (cardiovascular adj3 screen*).tw.<br>4. (population adj2 screen*).tw.

For peer review only

	5. (risk factor adj3 screen*).tw.<br>6. (opportunistic adj3 screen*).tw.<br>7. medical check*.tw.<br>8. general check*.tw.<br>9. periodic health exam*.tw.<br>10. annual exam*.tw.<br>11. annual review*.tw.<br>12. NHSHC.tw.<br>13. periodic medical examination/<br>14. 1 or 2 or 3 or 4 or 5 or 6 or 7 or 8 or 9 or 10 or 11 or 12 or 13<br>15. cardiovascular adj3 prevention.tw.<br>16. (primary care or general practice or primary healthcare).tw<br>17. 15 and 16<br>18. cardiovascular disease/ AND primary prevention/<br>19. 17 or 18<br>20. 14 or 19
Ovid HMIC	1 "health check*".af.<br>2 health checks/<br>3 (cardiovascular or vascular or heart or diabetes or stroke).af.<br>4 (screen* or risk).af.<br>5 3 AND 4<br>6 1 OR 2 or 5<br>7 cardiovascular adj3 prevention.tw.<br>8 (primary care or general practice or primary healthcare).tw<br>9 7 and 8<br>10 Cardiovascular diseases/ AND exp preventive medicine/<br>11 9 or 10<br>12 6 or 11
EBSCO CINAHL	S10 S1 OR S2 OR S9<br>S9 S5 OR S8<br>S8 S6 AND S7<br>S7 (MH "Preventive Health Care+")<br>S6 (MH "Cardiovascular Diseases+")<br>S5 S3 AND S4<br>S4 "primary care" or "general practice" or "primary healthcare"<br>S3 TX cardiovascular N3 prevention<br>S2 (diabetes N3 screen*) OR (cardiovascular N3 screen*) OR (population N2 screen*) OR (risk factor N3 screen*) OR (opportunistic N3 screen*) OR “medical check*” OR “general check*” OR “periodic health exam*” OR “annual exam*” OR "annual review*" OR NHSHC<br>S1 health check*
EBSCO Global Health	S10 S6 OR S19 OR S3 Limiters - Publication Year: 2016<br>S9 S7 AND S8<br>S8 DE "preventive medicine"<br>S7 DE "cardiovascular diseases"<br>S6 S4 AND S5<br>S5 "primary care" or "general practice" or "primary healthcare"

	S4 TX cardiovascular N3 prevention<br>S3 S1 OR S2<br>S2 (diabetes N3 screen*) OR (cardiovascular N3 screen*) OR (population N2 screen*) OR (risk factor N3 screen*) OR (opportunistic N3 screen*) OR “medical check*” OR “general check*” OR “periodic health exam*” OR “annual exam*” OR "annual review*" OR NHSHC<br>S1 health check*
HDAS PsycInfo	1 "health check*".af.<br>2 PHYSICAL EXAMINATION/<br>3 HEALTH SCREENING/<br>4 "diabetes screen*".af<br>5 "cardiovascular screen*".af<br>6 "population screen*".af<br>7 ("opportunistic* screen*" OR "risk factor screen*").af<br>8 ("medical check*" OR "general check*" OR "periodic health exam*" OR "annual exam*" OR "annual review*" OR NHSHC).af<br>9 1 OR 2 OR 3 OR 4 OR 5 OR 6 OR 7 OR 8<br>10 cardiovascular.ti,ab<br>11 prevention.ti,ab<br>12 10 AND 11<br>13 CARDIOVASCULAR DISORDERS/<br>14 PREVENTIVE MEDICINE/<br>15 13 AND 14<br>16 12 OR 15<br>17 9 OR 16
Web of Science, Science Citation Index	“health check*” OR “diabetes screen*” OR “cardiovascular screen*” OR “population screen*” OR “risk factor screen*” OR “Opportunistic screen*” OR “medical check*” OR “general check*” OR “periodic health exam*” OR “annual exam*” OR “annual review*” OR NHSHC<br>OR<br>(Cardiovascular NEAR/3 prevention) AND (“primary care” OR “general practice” OR “primary healthcare”)<br>Limit to: England, Scotland, Wales, North Ireland
Cochrane Library (Wiley)	#1 "health check*"<br>#2 (diabetes next/3 screen*) or (cardiovascular next/3 screen*) or (population next/2 screen*) or (opportunistic next/2 screen*) or ("risk factor" next/3 screen*) or "medical check*" or "general check*" or "periodic health exam*" or "annual exam*" or "annual review*" or NHSHC<br>#3 cardiovascular adj3 prevention.tw.<br>#4 (primary care or general practice or primary healthcare).tw<br>#5 #3 and #4<br>#6 MeSH descriptor: [Cardiovascular Diseases] this term only<br>#7 MeSH descriptor: [Primary Prevention] explode all trees<br>#8 #6 and #7<br>#9 #5 or #8<br>#10 #1 or #2 or #9

For peer review only

NHS Evidence	“health check*” OR cardiovascular prevention primary care
TRIP database	“health check*” OR cardiovascular prevention primary care
Google Scholar	"nhs health check"<br>cardiovascular “health check”<br>cardiovascular prevention “primary care”
Google	"nhs health check"<br>cardiovascular prevention “primary care”<br>cardiovascular “health check”
Clinical trials.gov and ISRCDN registry	“health check”

For peer review only

PRISMA 2009 Checklist

Section/topic	#	Checklist item	Reported on page #
**TITLE**			
Title	1	Identify the report as a systematic review, meta-analysis, or both.	1
**ABSTRACT**			
Structured summary	2	Provide a structured summary including, as applicable: background; objectives; data sources; study eligibility criteria, participants, and interventions; study appraisal and synthesis methods; results; limitations; conclusions and implications of key findings; systematic review registration number.	2
**INTRODUCTION**			
Rationale	3	Describe the rationale for the review in the context of what is already known.	4
Objectives	4	Provide an explicit statement of questions being addressed with reference to participants, interventions, comparisons, outcomes, and study design (PICOS).	4
**METHODS**			
Protocol and registration	5	Indicate if a review protocol exists, if and where it can be accessed (e.g., Web address), and, if available, provide registration information including registration number.	4
Eligibility criteria	6	Specify study characteristics (e.g., PICOS, length of follow-up) and report characteristics (e.g., years considered, language, publication status) used as criteria for eligibility, giving rationale.	5
Information sources	7	Describe all information sources (e.g., databases with dates of coverage, contact with study authors to identify additional studies) in the search and date last searched.	5
Search	8	Present full electronic search strategy for at least one database, including any limits used, such that it could be repeated.	Appendix 1
Study selection	9	State the process for selecting studies (i.e., screening, eligibility, included in systematic review, and, if applicable, included in the meta-analysis).	5
Data collection process	10	Describe method of data extraction from reports (e.g., piloted forms, independently, in duplicate) and any processes for obtaining and confirming data from investigators.	5/6
Data items	11	List and define all variables for which data were sought (e.g., PICOS, funding sources) and any assumptions and simplifications made.	Table 1 and 3
Risk of bias in individual studies	12	Describe methods used for assessing risk of bias of individual studies (including specification of whether this was done at the study or outcome level), and how this information is to be used in any data synthesis.	5
Summary measures	13	State the principal summary measures (e.g., risk ratio, difference in means).	5/6
Synthesis of results	14	Describe the methods of handling data and combining results of studies, if done, including measures of consistency (e.g., I^2) for each meta-analysis.	5

For peer review only

PRISMA 2009 Checklist

Section/topic	#	Checklist item	Reported on page #
Risk of bias across studies	15	Specify any assessment of risk of bias that may affect the cumulative evidence (e.g., publication bias, selective reporting within studies).	5/6
Additional analyses	16	Describe methods of additional analyses (e.g., sensitivity or subgroup analyses, meta-regression), if done, indicating which were pre-specified.	N/A
**RESULTS**			
Study selection	17	Give numbers of studies screened, assessed for eligibility, and included in the review, with reasons for exclusions at each stage, ideally with a flow diagram.	6 and Fig 1
Study characteristics	18	For each study, present characteristics for which data were extracted (e.g., study size, PICOS, follow-up period) and provide the citations.	Table 1 and 2
Risk of bias within studies	19	Present data on risk of bias of each study and, if available, any outcome level assessment (see item 12).	Table 2
Results of individual studies	20	For all outcomes considered (benefits or harms), present, for each study: (a) simple summary data for each intervention group (b) effect estimates and confidence intervals, ideally with a forest plot.	Table 1 and 3
Synthesis of results	21	Present results of each meta-analysis done, including confidence intervals and measures of consistency.	N/A
Risk of bias across studies	22	Present results of any assessment of risk of bias across studies (see Item 15).	N/A
Additional analysis	23	Give results of additional analyses, if done (e.g., sensitivity or subgroup analyses, meta-regression [see Item 16]).	N/A
**DISCUSSION**			
Summary of evidence	24	Summarize the main findings including the strength of evidence for each main outcome; consider their relevance to key groups (e.g., healthcare providers, users, and policy makers).	12/13
Limitations	25	Discuss limitations at study and outcome level (e.g., risk of bias), and at review-level (e.g., incomplete retrieval of identified research, reporting bias).	13
Conclusions	26	Provide a general interpretation of the results in the context of other evidence, and implications for future research.	13/14
**FUNDING**			
Funding	27	Describe sources of funding for the systematic review and other support (e.g., supply of data); role of funders for the systematic review.	14/15

From: Moher D, Liberati A, Tetzlaff J, Altman DG, The PRISMA Group (2009). Preferred Reporting Items for Systematic Reviews and Meta-Analyses: The PRISMA Statement. PLoS Med 6(6): e1000097. doi:10.1371/journal.pmed1000097

For more information, visit: www.prisma-statement.org.

For peer review only - http://bmjopen.bmj.com/site/about/guidelines.xhtml
